# Identification of lncRNA dual targeting PD-L1 and PD-L2 as a novel prognostic predictor for gastric cancer

**DOI:** 10.3389/fonc.2024.1341056

**Published:** 2024-10-25

**Authors:** Li-Na Zhang, Jiong-Yu Chen, Yu-Xin Liu, Yue Zhang, Liang-Li Hong, Xin-Xin Li, Shu-Hui Liu, Shu-Qin Chen, Lin Peng, Yi-Teng Huang

**Affiliations:** ^1^ Department of Pathology, The First Affiliated Hospital of Shantou University Medical College, Shantou, Guangdong, China; ^2^ Central Laboratory, Cancer Hospital of Shantou University Medical College, Shantou, Guangdong, China; ^3^ Health Care Center, The First Affiliated Hospital of Shantou University Medical College, Shantou, Guangdong, China; ^4^ Department of General Surgery, The First Affiliated Hospital of Shantou University Medical College, Shantou, Guangdong, China; ^5^ Department of Pathology, Shantou University Medical College, Shantou, Guangdong, China; ^6^ Biological Specimen Repository, Cancer Hospital of Shantou University Medical College, Shantou, Guangdong, China

**Keywords:** gastric carcinoma, LINC01094, PD-L1, PD-L2, tumor immune microenvironment

## Abstract

**Background:**

Although breakthroughs have been achieved in gastric cancer (GC) therapy with immune checkpoint inhibitors (ICIs) targeting programmed death-1 (PD-1) and programmed death-ligand 1 (PD-L1), the acquisition of high response rate remains a huge challenge for clinicians. It is imperative to identify novel biomarkers for predicting response to immunotherapy and explore alternative therapeutic strategy for GC.

**Methods:**

The transcriptomic profiles and clinical information of GC patients from The Cancer Genome Atlas (TCGA)-stomach adenocarcinoma (STAD) database was used to screen differentially expressed lncRNAs between the tumor specimens and the paracancerous tissues. The TargetScan, miRDB and miRcode database were then utilized to construct competing endogenous RNA (ceRNA) networks and identify pivotal lncRNAs. An independent dataset from GEO (GSE70880) and 23 pairs of GC specimens of our cohort were subsequently performed for external validity. The relationship between clinical variables and gene expression were evaluated by Kruskal–wallis test and Wilcoxon signed-rank. The prognostic value of the candidate genes was assessed using Kaplan-Meier analysis and Cox regression models. CIBERSORT and Gene set enrichment analysis (GSEA) were used to determine immune cell infiltration. Gastric adenocarcinoma AGS cells and human embryonic kidney 293T (HEK293T) cells with knockdown of LINC01094 were generated by siRNA transfection, followed by detecting the alteration of the target miRNA and PD-L1/PD-L2 by RT-qPCR. Besides, the interaction between lncRNA and the miRNA–PD-L1/PD-L2 axis were verified by dual luciferase reporter assay.

**Results:**

Twenty-two intersecting lncRNAs were identified to be PD-L1/PD-L2-related lncRNAs and LINC01094–miR-17-5p–PD-L1/PD-L2 was constructed as a potential ceRNA network. LINC01094 was increased in tumor specimens than adjacent normal samples and was positively associated with advanced tumor stages and EBV and MSI status. Furthermore, LINC01094 expression was an independent risk factor for poor overall survival (OS) in GC patients. CD8^+^ T cell exhaustion-related genes were enriched in high-LINC01094 tissues and high-PD-L2 group. A strong positive association of LINC01094 expression was established with M2 macrophages, IL-10^+^ TAM, as well as PD-L1 and PD-L2 levels, therefore a LINC01094–miR-17-5p–IL-10 network was proposed in macrophages. Using the exoRBase database, LINC01094 was assumed in blood exosomes of GC patients The results of knockdown experiments and luciferase reporter assays revealed that LINC01094 interacted with miR-17-5p and served as a miRNA sponge to regulate the expression of PD-L1 and PD-L2.

**Conclusion:**

LINC01094 dually regulates the expression of PD-L1 and PD-L2 and shapes the immunosuppressive tumor microenvironment via sponging miR-17-5p. LINC01094 may serve as a potential prognostic predictor and therapeutic target in GC.

## Graphical Abstract

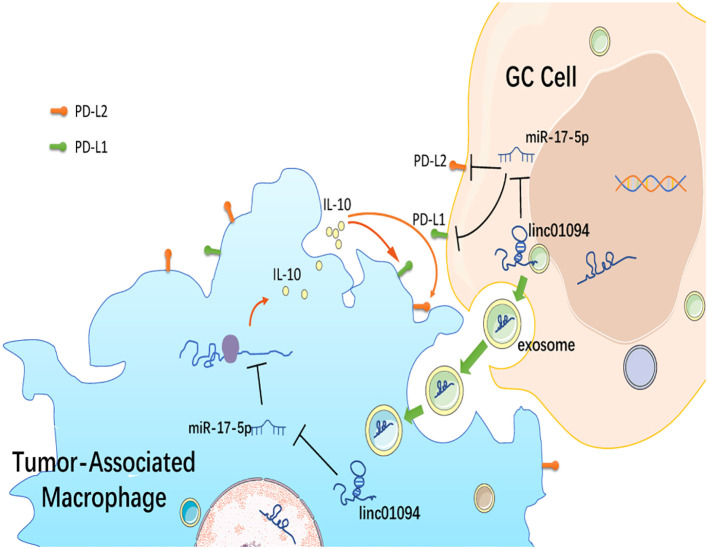

## 1 Introduction

Gastric cancer (GC) remains the third deadly malignant disease because most patients are diagnosed at advanced stage and effective treatments are lacking (1). Although the newly developed immune checkpoint inhibitors (ICIs) have paved the way to a new era in cancer therapy (2), the objective response rate of advanced GC patients with ICI does not exceed 12% (3). Recent randomized controlled trials for nivolumab and pembrolizumab, the ICIs targeting PD-1 and PD-L1 respectively, showed the inconsistent efficacy in GC patients (4). Although CAR-T cell therapy has been developed as a promising option for solid tumor treatment in combination with ICI, systemic administration of the antibody was indicated to have adverse effects and high cost inevitably (5). Hence, the implementation of novel treatment approaches is imperative to improve the outcomes of GC.

PD-L1 expression within the tumor microenvironment (TME) has been associated with response to anti-PD-1 therapies in various malignancies, including GC (6–8). PD-L1 was then suggested as one of the biomarkers for identification of the GC patients that most likely benefit from immunotherapy and targeted therapy (9). Nevertheless, the phase 3 KEYNOTE-062 trial reported conflicting results for the use of PD-L1 as a prognostic or predictive biomarker in gastric adenocarcinoma, since pembrolizumab plus chemotherapy did not improve OS or PFS in patients who had a PD-L1 positivity ≥ 1 or ≥ 10. Therefore, identifying more robust predictive biomarkers for checkpoint-based immunotherapy in GC and exploring new blockade therapeutic approaches are needed to optimize treatment for GC.

PD-L2, commonly expressed on tumor cells, macrophages, and dendritic cells (10), is another ligand of the PD-1 receptor, and has approximately 2-6-fold higher affinity compared to PD-L1 (11). Overexpression of PD-L2 has been found in many solid malignancies and is independently associated with clinical response to ICI in a variety of tumor types (12–16). Recent studies signify the predictive, prognostic and therapeutic value of PD-L2 for GC immunotherapy, as evidenced by the association of PD-L2 and PD-L2 mRNA expression with tumor progression and poor survival, as well as the infiltration of myeloid dendritic cells, CD4^+^ T-cells and CD8^+^ T-cells (17–19). PD-L1 is detected in about 50% of GC patients (20, 21), whereas 28.4% of GC patients express tumor-cell PD-L2, and 16.0% GC patients co-express PD-L1 and PD-L2 (17). Wu et al. indicated that the positive expression rates of PD-L1 and PD-L2 in tumor cells of Helicobacter pylori- and Epstein-Barr virus (EBV)-associated GC were 40.3% and 53.8%, respectively (22). These findings suggest that monotherapy targeting PD-L1 or PD-L2 alone may not be sufficient to show significant benefits for GC patients. Recently, Fan et al. indicated that PD-L1 and PD-L2 are co-regulated by lncRNA PCED1B−AS1 via sponging hsa−miR−194−5p to induce immunosuppression in hepatocellular carcinoma (23). However, systematic analysis of the co-regulatory mechanism for both PD-L1 and PD-L2 expression in GC is scarce.

Competitive endogenous RNA (ceRNA) regulatory networks have been implicated in cancer pathogenesis and progression (24–26), as well as shaping the tumor immune landscape (27, 28), among which long non-coding RNAs (lncRNAs) exert huge influence. It has been documented that lncRNAs regulate multiple target genes simultaneously via sponging miRNAs, and lncRNA/miRNA-based therapeutics have been suggested to be a potential strategy for cancer therapy (29–31). Thus, there is a need to explore the potential interactions of PD-L1/PD-L2 with lncRNA and miRNA. In this study, we performed a comprehensive analysis to identify lncRNA-associated ceRNA networks involved in regulation of PD-L1/PD-L2 and immune cell infiltration in GC. We then performed immune cell infiltration analysis to explore the function of this axis, which may help in developing effective prognostic markers and novel strategies to boost the development of immune checkpoint blockade therapy for GC.

## 2 Methods and material

### 2.1 Data collection and processing

We collected the transcriptome data in counts format of 407 STAD samples from TCGA (https://portal.gdc.cancer.gov/), including 375 tumor tissue samples and 32 paracancerous tissue samples. The expression matrix was standardized using log2 (CPM + 1) values. Clinical information including age at diagnosis, sex, TNM stage, pathological grade, molecular subtype and survival time were downloaded from TCGA Pan-Cancer Clinical Data Resource (TCGA-*CDR*). The GSE70880 dataset (n = 40), comprising mRNA expression microarray data and lncRNA microarray data from 20 gastric tissues and 20 paracancerous tissues based on the GPL19748 platform (Agilent-038314 CBC Homo sapiens lncRNA + mRNA microarray V2.0 (Probe Name version)) was also downloaded.

### 2.2 Identification of PD-L1/PD-L2-related differentially-expressed lncRNAs

We applied the “limma” R package for the entire lncRNA data to identify the differentially-expressed lncRNAs (DE lncRNAs) between tumor and paracancerous specimens, with screening criteria set at |log fold change (logFC)| > 1 and adjusted *p*-value < 0.05. The prognostic significance of DE lncRNAs was validated using an external dataset obtained from the GEO database (GSE70880). Then, Spearman’s Rank correlation was performed to assess the correlation of candidate DE lncRNAs with PD-L1 and PD-L2 DE lncRNAs with coefficient > 0.4 and *p*-value < 0.001. Subsequently, the intersecting DE lncRNAs for PD-L1 and PD-L2 were obtained by VennDiagram R package.

### 2.3 Target prediction of mRNA and lncRNA

Targetscan (http://targetscan.org/) and miRDB (http://mirdb.org/miRDB/) were used for mRNA target‐gene prediction, and the intersection of results from the two software was selected as the array of predicted target genes of mRNA. Then, target predication for lncRNAs were carried out using miRcode (http://http://www.mircode.org/) and RNAhybrid (https://bibiserv.cebitec.uni-bielefeld.de/rnahybrid). Only miRNA target genes that were identified by all three methods were selected as the ceRNA prediction result.

### 2.4 Construction of the ceRNA network

A ceRNA network was constructed based on ceRNA theory as follows: (1) lncRNA-mRNA pairs with SCC > 0.5 and *p*-value < 0.001 were selected as target pairs, (2) if both the lncRNA and mRNA were negatively co-expressed with a common miRNA, this lncRNA-miRNA-mRNA group was identified as a co-expressed competing triplet (32), and (3) the binding sites of lncRNA-miRNA and miRNA-mRNA were predicted by Targetscan, miRDB, miRcode and RNAhybrid. The PD-L1/PD-L2-related ceRNA networks were then visualized by using Cytoscape 3.6.1 software (33).

### 2.5 Validation in clinical samples

A total of 23 pairs of specimens, including gastric adenocarcinoma tissues and adjacent normal tissues were recruited from Cancer Hospital of Shantou University Medical College. Total RNA was extracted from the tissues with TRIzol reagent (Invitrogen, Carlsbad, CA, USA) according to the manufacturer’s instructions. The HiScript III 1st Strand cDNA Synthesis Kit (#R312, Vazyme, Nanjing, China), The HiScript III SuperMix for qPCR (+gDNA wiper) cDNA Synthesis Kit (#R323, Vazyme) and ChamQ Universal SYBR qPCR Master Mix (#Q711, Vazyme) were utilized to measure the expression of lncRNA and mRNA, of which the primers sequences were listed in **Supplementary Table 1**. The mean value of relative gene concentrations in each triplicate were calculated using 2^−ΔCt^ by normalizing with β-actin. The research protocol was approved by the Ethics Committee of the Cancer Hospital of Shantou University Medical College (2022048) and written informed consent was obtained from all participants.

### 2.6 Cell transfection and reverse transcription-quantitative polymerase chain reaction

The siRNA specifically targeting LINC01094 (siLINC01094) and its corresponding control (siNC) were designed and purchased from Gene Pharma Co. (Shanghai, China) and transfected using Lipofectamine 3000 (Thermo Fisher Scientific, USA). The sequence information for the above vectors or oligonucleotides was presented in **Supplementary Table 2**. Total RNA was extracted from the cells using TRIzol reagent (Invitrogen, Carlsbad, CA, USA). The complementary DNA for mRNA analysis was synthesized using the HiScript III SuperMix for qPCR (+gDNA wiper) cDNA Synthesis Kit (#R323, Vazyme), The complementary DNA for lncRNA analysis was synthesized using the HiScript III 1st Strand cDNA Synthesis Kit (#R312, Vazyme). RT-qPCR was conducted on ABI7500 Real-time PCR System (Applied Biosystems, Foster City, CA, USA) to detect the relative expression level of LINC01094, PD-L1 and PD-L2. The results were calculated using 2^ΔΔC^_T_ method. The primer sequences are listed in **Supplementary Table 1.**

### 2.7 Dual luciferase reporter assay

The putative binding sites between LINC01094 and miR-17-5p, miR-17-5p and PD-L1, and miR-17-5p and PD-L2 were predicted using TargetScan and RNAhybrid (**Supplementary Figure 1**). The HEK293T cells were used for luciferase reporter assay. The fragments including the 3’UTR regions (3’UTR-WT) or mutant 3’UTR regions (3’UTR-Mut) of LINC01094, and PD-L1 and PD-L2 were inserted into pmirGLO luciferase vector respectively (GenePharma, Shanghai, China), followed by transfection of miR-17-5p mimic and the mimic control. At 48 h posttransfection, cells were collected and detected for the firefly and Renilla luciferase activities using Dual Luciferase Reporter Assay kit (Yeasen Technology, Shanghai, China) by multimode reader (BioTek Synergy H1, USA).

### 2.8 Clinicopathological and survival analysis

Patients were divided into high-level and low-level groups according to the median expression level. Wilcoxon rank‐sum and Kruskal-Wallis tests were used to examine the link between the candidate lncRNA and clinical characteristics. The Kaplan-Meier analysis with log-rank test and Cox regression models were performed to assess the prognostic value of candidate lncRNAs for GC. Survival curves were visualized using the R package “survival” (http://cran.r‐project.org/web/packages/survival/index.html).

### 2.9 Immune cell infiltration analysis

CIBERSORT (cell-type identification by estimating relative subsets of RNA transcripts) is a bioinformatics algorithm to calculate cell composition from gene expression profiles of complex tissues (34). Combination of CIBERSORT and LM22 (leukocyte signature matrix) is used to calculate the content of 22 types of human leukocyte subsets. We used the R package “CIBERSORT” to calculate the number of immune cells in each sample of the cohort in TCGA-STAD. Immune score represents the content of immune cells. The optimal cutoff point for immune cell expression was determined by the R package survminer (https://CRAN.R-project.org/package=survminer). Furthermore, the Wilcoxon test was used to compare the difference in cell content between high- and low-PD-L2 expression groups.

### 2.10 IL-10^+^ TAM infiltration and CD8^+^ T cell function analysis

Gene set variation analysis (GSVA) was used to calculate sample-wise enrichment scores for 12 IL-10^+^ TAM-associated gene signatures (35). Then, the correlation of IL-10^+^ TAMs with LINC01094 and PD-L1, as well as PD-L2 expression, was evaluated by correlation modules. To explore CD8^+^ T cell function signaling pathway enrichment, GSEA (36) was performed between low and high LINC01094 expression groups using the R package “clusterProfiler” (37) and “GSEAbase”. A ranked list was prepared using the log2 fold change of all expressed genes. IL-10^+^ TAM-associated gene and custom exhausted CD8^+^ T cell gene set were listed in **Supplementary Table 3** (38).

### 2.11 Exosome-derived lncRNA analysis

ExoRBase is a web-accessible database, which provides circular RNA, lncRNA and mRNA information from RNA-seq data analyses of human exosomes (31). We searched the profile of LINC01094 expression levels in cerebrospinal fluid, urine and blood of different cancers, and downloaded lncRNAs expression profiles from 9 GC blood samples.

### 2.12 Statistical analysis

R software (Version 3.6.0) and corresponding R packages were utilized for statistical analysis. Candidate prognostic factors with a *p*-value < 0.1 in univariate Cox regression analysis were incorporated into multivariate Cox regression analysis. Spearman’s correlation was performed to analyze correlations. For continuous variables, Student’s *t*-test (two tailed) was applied to assess intergroup differences for the normally distributed data. The Wilcoxon matched-pair signed-rank test was used to explore the difference in gene expression between.

## 3 Results

### 3.1 Basic characteristics of patients

**Figure 1:**
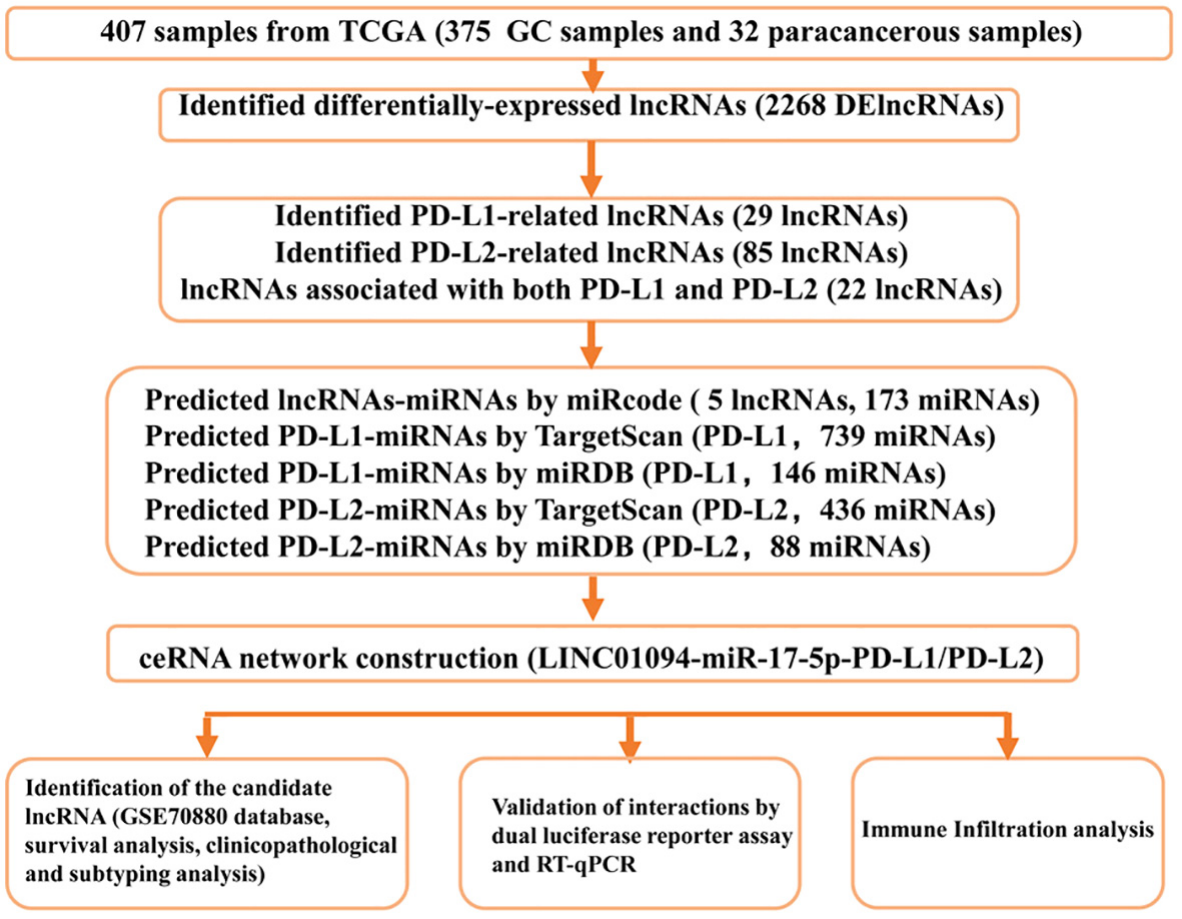
Study flowchart.

A flow diagram of this study is shown in **Figure 1**. In total, 375 microarray profiles obtained from the dataset in TCGA-STAD are available for analysis. The clinical characteristics of patients are summarized in **Supplementary Table 4.**

### 3.2 DElncRNAs in STAD with different PD-L1/PD-L2 expressions

**Figure 2:**
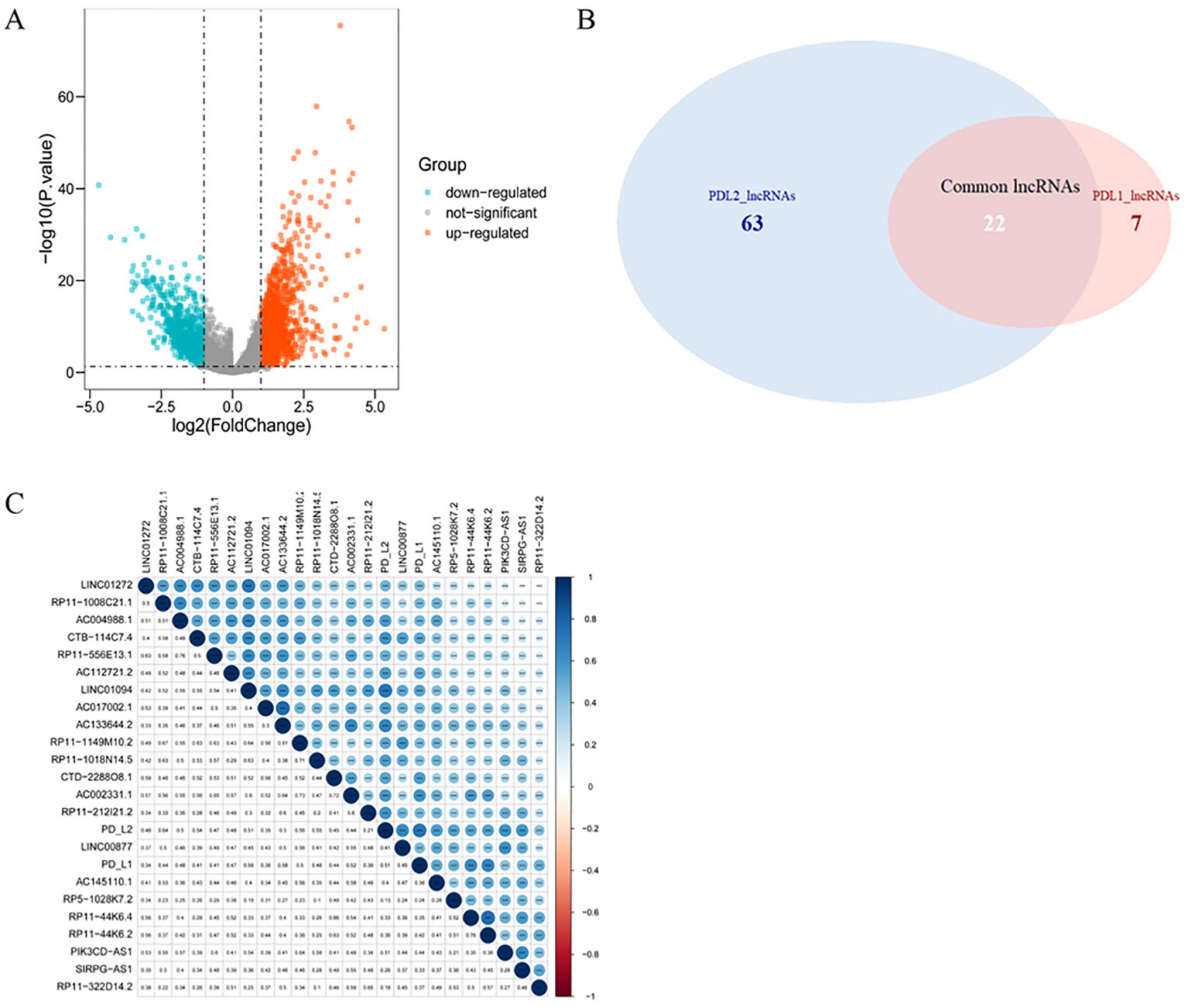
PD-L1- and PD-L2-co-associated DElncRNAs in GC. **(A)**, Volcano plots of differentially-expressed lncRNAs between GC and normal samples. Blue nodes represent down-regulated DElncRNAs, while orange nodes represent up-regulated DElncRNAs. **(B)**, Venn diagram showing the overlap for lncRNAs of PD-L1 and PD-L2. **(C)**, Correlation between PD-1/PD-L2 and their 22 co-related lncRNAs. Darker blue represents stronger correlation. * *p* = 0.05, ** *p* = 0.01, *** *p* = 0.001.

Compared with normal tissue, a total of 2268 lncRNAs were confirmed to be differentially expressed in GC samples. Specifically, 1377 lncRNAs were upregulated and 891 were downregulated (**Figure 2A**). Among them, 29 lncRNAs were positively correlated with the expression of PD-L1 and 85 of that had positive coefficients with PD-L2 (**Supplementary Table 5**). Subsequently, 22 intersecting lncRNAs from the two relevant lncRNA groups were identified to be PD-L1/PD-L2-related lncRNAs (**Figure 2B**), as displayed in **Figure 2C**.

### 3.3 Identification of a PD-L1/PD-L2-related lncRNA-mediated ceRNA network in GC

**Figure 3:**
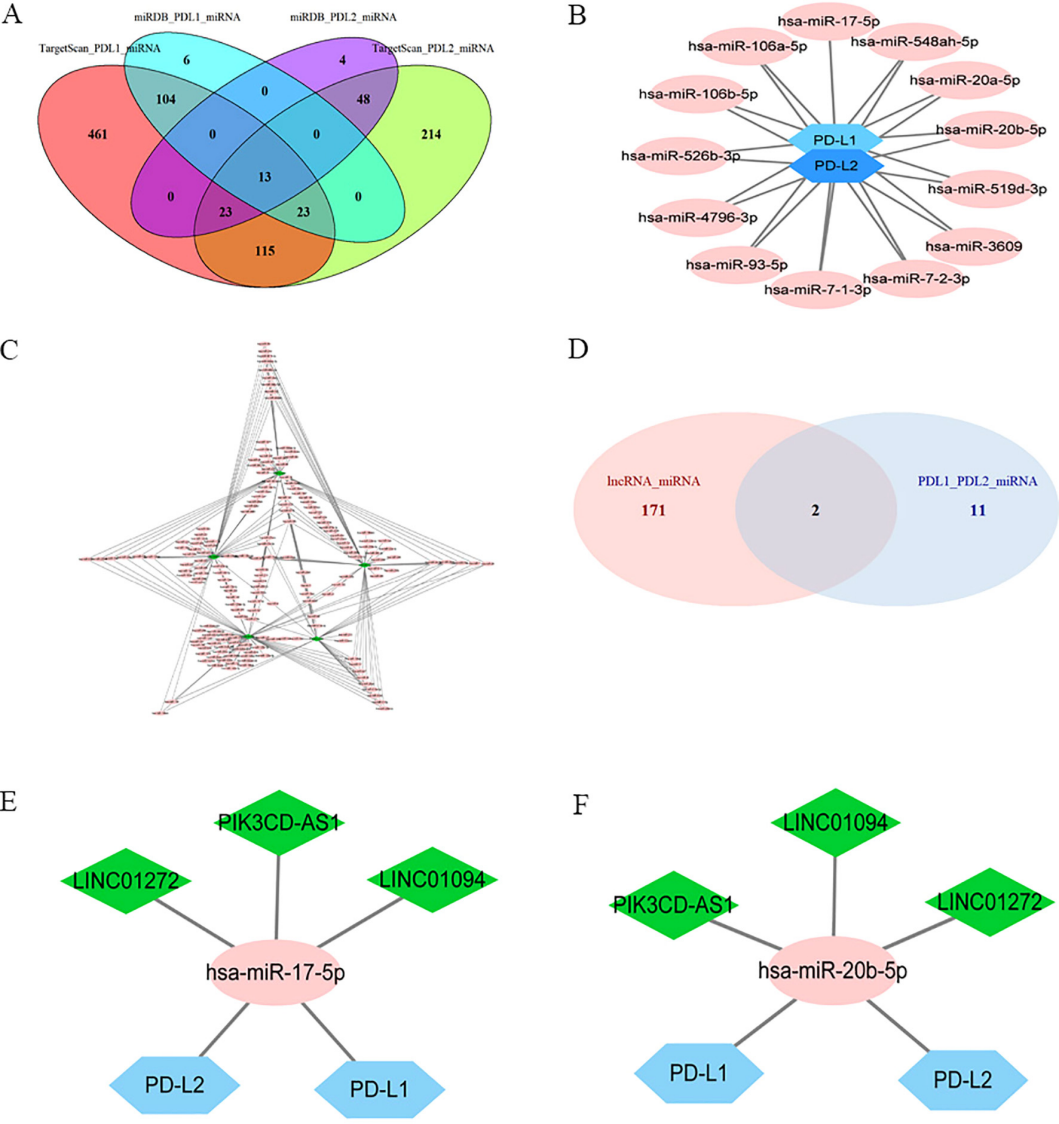
Identification of a PD-L1/PD-L2-related lncRNA-miRNA axis in GC. Ellipses denote miRNAs, diamonds denote lncRNAs, and hexagons denote mRNA. **(A)**, Venn diagram of PD-L1/PD-L2-interacting miRNAs by Targetscan and miRDB. **(B)**, PD-L1/PD-L2 targeted miRNAs. **(C)**, DElncRNAs targeted miRNAs. **(D)**, Venn diagram of common miRNAs of DElncRNAs, PD-L1 and PD-L2. **(E)**, Co-expression network of lncRNA–miR-17-5p–PD-L1/PD-L2. **(F)**, Co-expression network of lncRNA–miR-20b-5p–PD-L1/PD-L2.

Targetscan and miRDB were used to predict potential miRNA-PD-L1/PD-L2 relationships, and 13 miRNAs were selected as PD-L1/PD-L2 target genes as a result of the intersection of the two software (**Figures 3A, B**). The miRcode online website was used to predict the downstream target miRNAs of the 22 intersecting DElncRNAs. As a result, five lncRNAs (LINC01094, LINC01272, LINC00877, PIK3CD-AS1 and SIRPG-AS1) and their associated miRNAs were identified (**Figure 3C**). Then, two intersecting miRNAs (**Figure 3D**), namely miR-17-5p and miR-20b-5p, was identified between target miRNAs from DElncRNA–miRNA pairs (**Figure 3C**) and miRNAs–PD-L2/PD-L2 pairs (**Figure 3B**) by Venn diagram analysis. A network of three lncRNAs, two miRNAs and PD-L1/PD-L2 was constructed (**Figures 3E, F**).

As shown in **Figure 2C**, the correlations between LINC01094 and PDL1/PDL2 were 0.58 and 0.51, LINC01272 and PDL1/PDL2 were 0.34 and 0.46, PIK3CD-AS1 and PDL1/PDL2 were 0.44 and 0.51, respectively. Therefore, we choose LINC01094 with the highest correlation as the next step of research. Correlation analysis of ENCORI online website demonstrated that the correlation coefficient of LINC01094 with miR-17-5p and miR-20b-5p were -0.267 and -0.122 (**Supplementary Figure 2A-B**). Notably, miR-17-5p showed stronger correlation with PD-L2 than miR-20b-5p (**Supplementary Figure 2E-F**). Considering the interaction of miR-17-5p and PD-L1 has been previously described (39), miR-17-5p was filtrated as the potential node and a ceRNA network of LINC01094–miR-17-5p–PD-L1/PD-L2 was then constructed. Using the LncATLAS website, the intracellular localizations of LINC01094 was inspected to present in the cytoplasm, suggesting its role in gene expression regulation as expected (**Supplementary Figure 3**).

### 3.4 Validation of the upregulation of LINC01094 in GEO dataset and clinical samples

**Figure 4:**
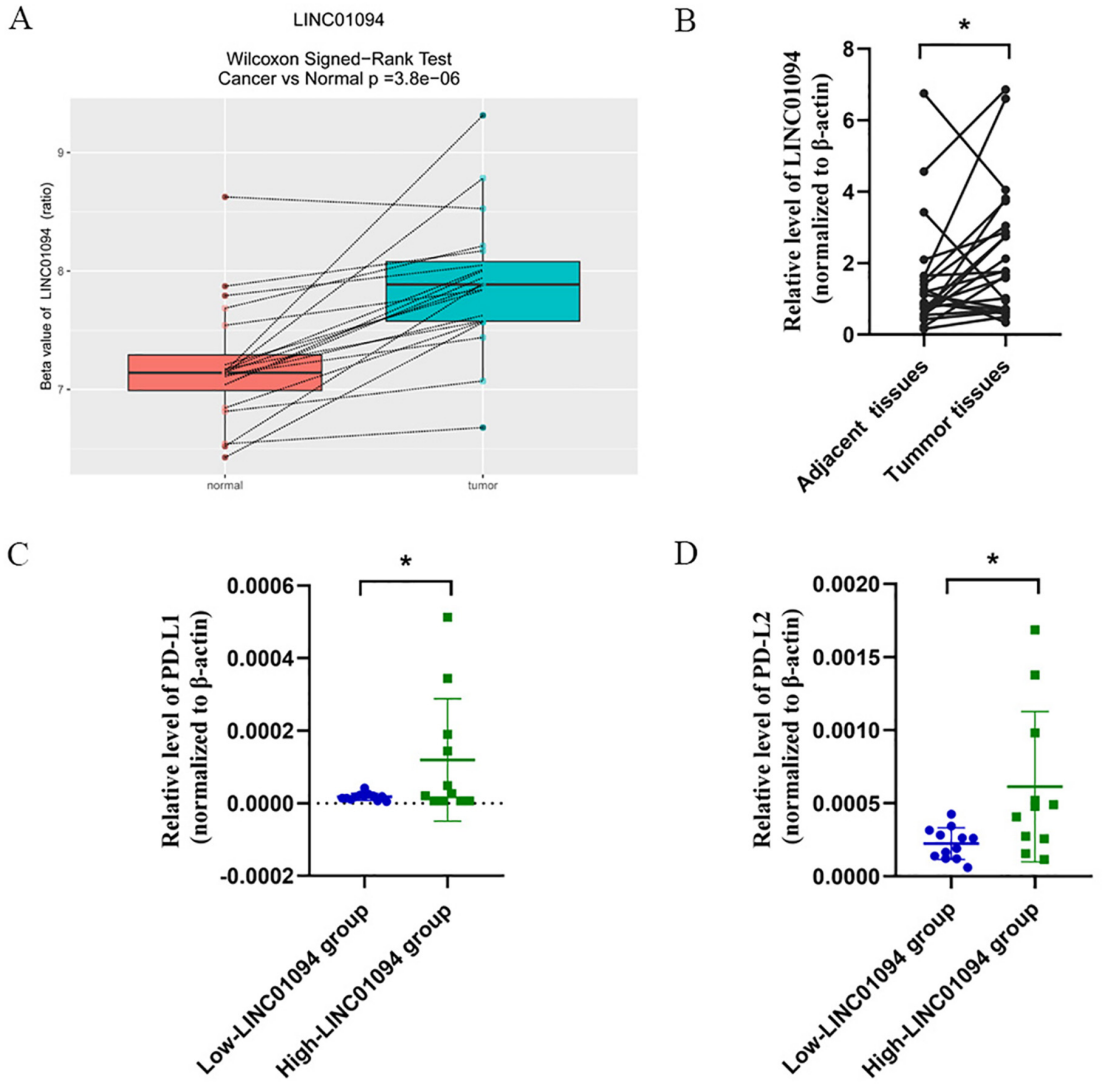
**(A)**, Boxplot of LINC01094 expression in 20 pair GC and adjacent tissues (GSE70880 data set). **(B)**, Relative LINC01094 expression detected by RT-qPCR in 23 paired gastric cancer and noncancerous tissues. Results are presented as 2^-ΔΔCt^ in tumor tissues relative to adjacent tissues. **(C, D)**, Relative expression levels of PD-L1 **(C)** and PD-L2 **(D)** in low and high LINC01094 expression groups in gastric tumors. *p < 0.05.

An independent dataset from GEO database (GSE70880) and twenty-three pairs of GC samples we collected were performed for external validity. As expected, LINC01094 was upregulated in GC tissues in comparison with the adjacent normal tissues in the GSE70880 datasets (**Figure 4A**). Similarly, the RT-qPCR results of our cohort showed LINC01094 levels were dramatically higher in tumor specimens than adjacent normal samples (**Figure 4B**). Furthermore, the expression levels of PD-L1 and PD-L2 were significantly higher in high-LINC01094 group (n = 11) than low-LINC01094 group (n = 12) (**Figures 4C, D**).

### 3.5 Validation of the regulatory interactions among LINC01094-miR-17-5p-PD-L1/PD-L2

**Figure 5:**
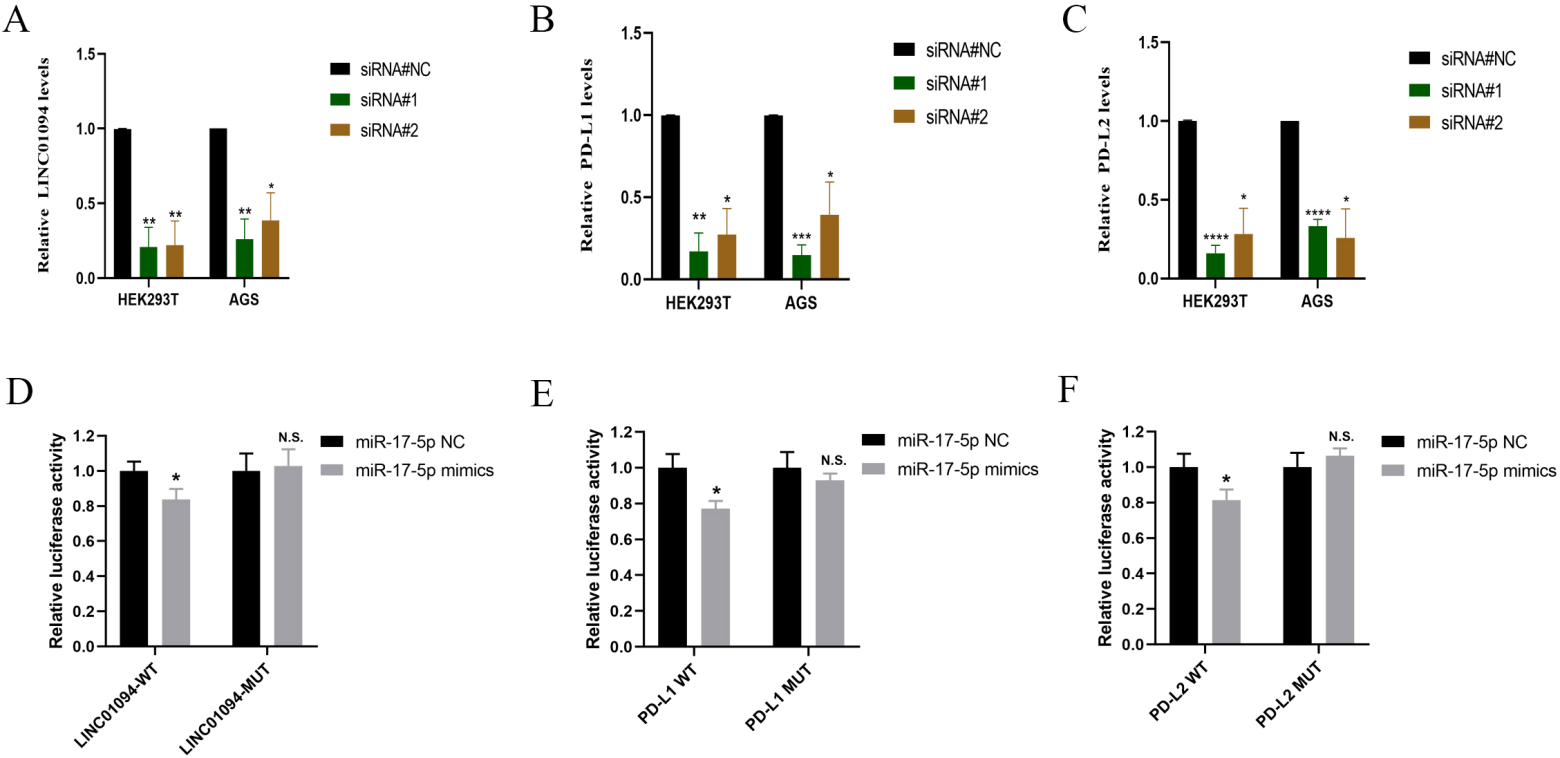
LINC01094 sponges miR-17-5p to enhance the expression of PD-L1 and PD-L2. A-C, The effect of LINC01094 knockdown on the relative mRNA expression of LINC01094 **(A)**, PD-L1 **(B)** and PD-L2 **(C)** in HEK-293T and AGS cells. D-F, The relative luciferase activities were determined between co-transfection with miR-17-5p mimic or miR-17-5p mimic-NC in HEK-293T cells, among which cells co-transfected with luciferase reporter gene driven by either the wild type of LINC01094 (LINC01094-WT) or the mutated LINC01094 (LINC01094 -MUT) were shown in **(D)**, cells co-transfected with reporter gene driven by either the wild-type or mutant 3′UTR of PD-L1 (PD-L1-WT, PD-L1-MUT) and PD-L2 (PD-L2-WT, PD-L2-MUT) were displayed in **(E, F)** respectively. The p value among groups was calculated by Student’s t-test. N.S. represents No significance, *p < 0.05, **p < 0.01, ***p < 0.001 and ****p < 0.0001.

In view of the hypothesis that LINC01094 functioned as a miR-17-5p decoy and miR-17-5p doubly targeted the 3’UTR of PD-L1 and PD-L2 in GC, RT-qPCR was performed to determine the alteration of PD-L1 and PD-L2 after LINC01094 silencing. As (**Figures 5A–C**) showed that, LINC01094, PD-L1 and PD-L2 mRNA expression was significantly reduced in AGS cells and HEK293T cells transfected with siLINC01094.

Subsequently, the luciferase reporter assay was used to verify the interaction between LINC01094 and miR-17-5p and PD-L1/PD-L2 in HEK293T. We introduced mutation into the potential binding sequences of LINC01094 and then showed that miR-17-5p mimics specifically suppressed the luciferase activity driven by wildtype, but not mutant LINC01094 sequences (*p* < 0.05, **Figure 5D**), demonstrating the direction interaction between these two non-coding RNA molecules. Furthermore, the luciferase activity driven by miR-17-5p mimic was significantly decreased compared to the control in PD-L1-3’URT-WT and PD-L2-3’URT-WT group (*p* < 0.05, **Figures 5E, F**), indicating that the prediction binding site of miR-17-5p strongly contributes to PD-L1 and PD-L2. These data suggested that LINC01094 could regulate both PD-L1 and PD-L2 expression by sponging miR-17-5p.

### 3.6 Upregulation of LINC01094 correlates with the clinicopathological characteristics, molecular subtypes and prognosis of GC

**Figure 6:**
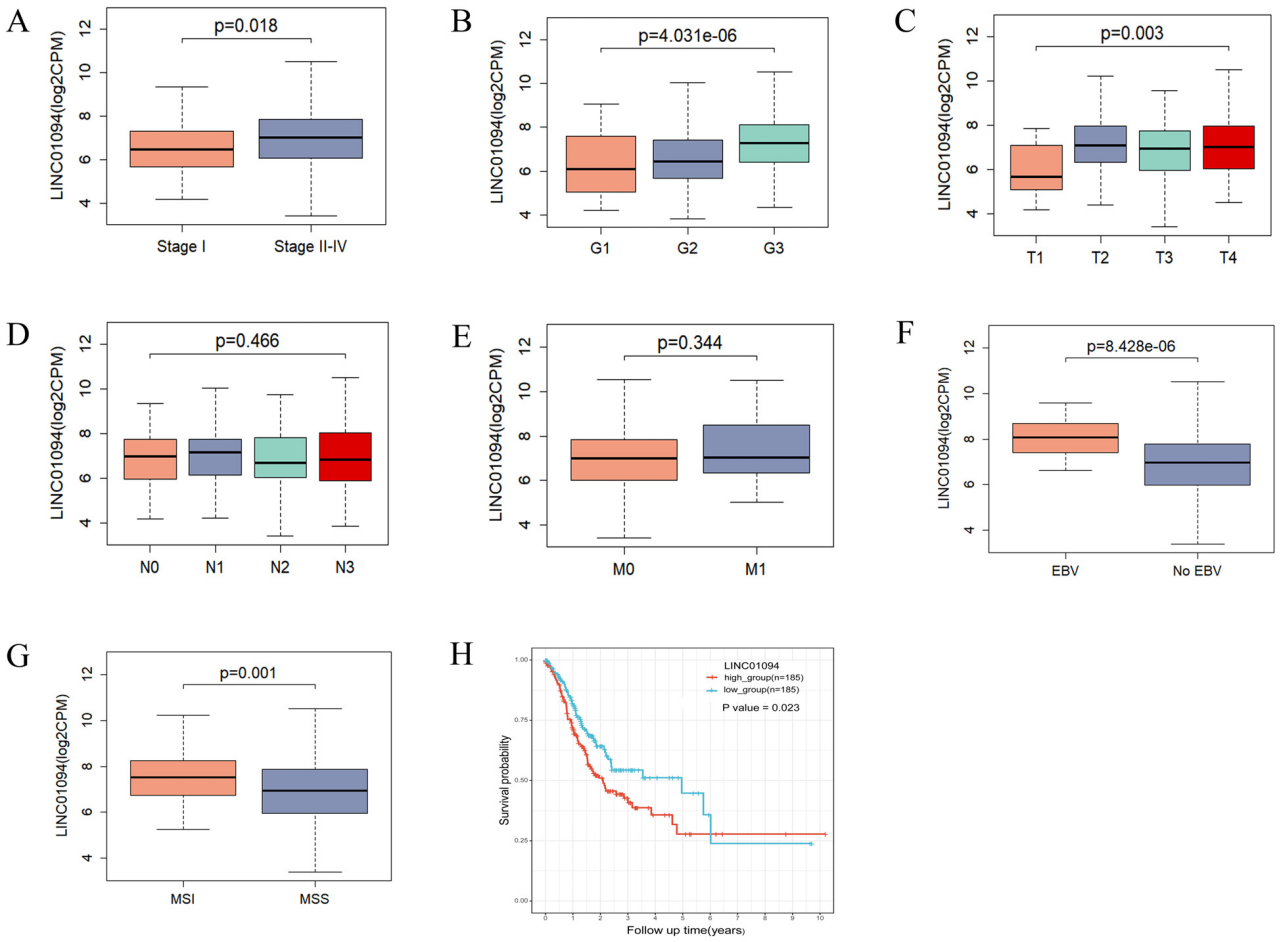
Correlations of LINC01094 with clinicopathological characteristics, molecular subtypes and prognosis of GC. **(A–E)**, Association analysis between LINC01094 levels and clinical stage **(A)**, histologic grade **(B)**, T stage **(C)**, N stage **(D)**, M stage **(E)**, **(F, G)**, Association of LINC01094 with molecular subtypes, EBV **(F)**, MSI **(G)**. **(H)**, Kaplan-Meier curves for the overall survival of patients in the high- and low LINC01094 expression groups in TCGA-STAD cohort.

Regarding the clinical value of the hub lncRNA, stratified analysis was performed to validate whether LINC01094 could distinguish clinicopathological characteristics, as well as survival differences in GC patients. Clinical feature analysis showed that GC patients with advanced clinical stage (*p* = 0.018), pathological grade (*p* = 4.03e-06) and T stage (*p* = 0.003) had higher levels of LINC01094 (**Figures 6A–C**), while no significant correlation was found for lymph node metastasis or distant metastasis (**Figures 6D, E**, *p* > 0.05). Additionally, higher LINC01094 levels were found in the Epstein-Barr virus (EBV) and micro satellite instability (MSI) subtypes (all *p* ≤ 0.001, **Figures 6F, G**).

Kaplan-Meier curves indicated that GC patients with higher LINC01094 levels had worse survival (log-rank, *p* = 0.023; **Figure 6H**). Other variables, including age, gender, clinical stage, histological grade, molecular subtypes and LINC01094 expression level were assessed using univariate Cox regression analysis. Afterwards, those with a *p*-value < 0.05 were incorporated into the multivariate Cox regression model (**Table 1**). Ultimately, LINC01094 was identified as an independent prognostic factor for GC patients (HR = 1.74, 95% CI = 1.09-2.77, *p* = 0.02) (**Table 1**).

**Table 1:** Univariate and multivariate Cox analysis of OS related prognostic factors in patients with GC.

Characterstics	Univartate analysis				Multivartate analysis		
	OR	95%CI	*p*		OR	95%CI	*p*
LINC01094							
low	1.00						
high	1.59	1.05 - 2.41	0.03		1.74	1.09-2.77	0.02
**Age**	1.02	1.00 - 1.04	0.12		–	–	–
Sex							
male	1.00						
female	0.69	0.44 - 1.07	0.10		–	–	–
Clinical stage							
I	1.00						
II	1.51	0.71 - 3.22	0.29		0.80	0.24-2.75	0.73
III	2.83	1.38 - 5.82	<0.001		1.21	0.23-6.28	0.82
IV	5.24	2.10 - 13.08	<0.001		2.25	0.40-12.80	0.36
T stage							
T1	1.00						
T2	4.33	0.93 - 20.13	0.06		3.39	0.65-17.64	0.15
T3	6.53	1.46 - 29.17	0.01		4.06	0.62-26.70	0.14
T4	5.91	1.29 - 26.96	0.02		2.59	0.37-18.16	0.34
M stage							
M0	1.00						
M1	2.06	0.91 - 4.68	0.08		–	–	–
N stage							
N0	1.00						
N1	2.07	1.15 - 3.71	0.01		1.58	0.70-3.57	0.27
N2	1.84	0.99 - 3.45	0.06		1.36	0.49-3.79	0.55
N3	3.71	1.99 - 6.93	<0.001		2.38	0.85-6.71	0.10
Histologic grade							
G1	1.00						
G2	2.23	0.45 - 10.90	0.32		–	–	–
G3	2.90	0.60 - 13.96	0.18		–	–	–
MSI							
No	1.00						
Yes	0.73	0.40-1.30	0.28	–	–	–	–
EBV							
No	1.00						
Yes	0.77	0.34-1.77	0.54	–	–	–	–

### 3.7 LINC0104–PD-L1/PD-L2 axis correlates with immune cell infiltration in GC

**Figure 7:**
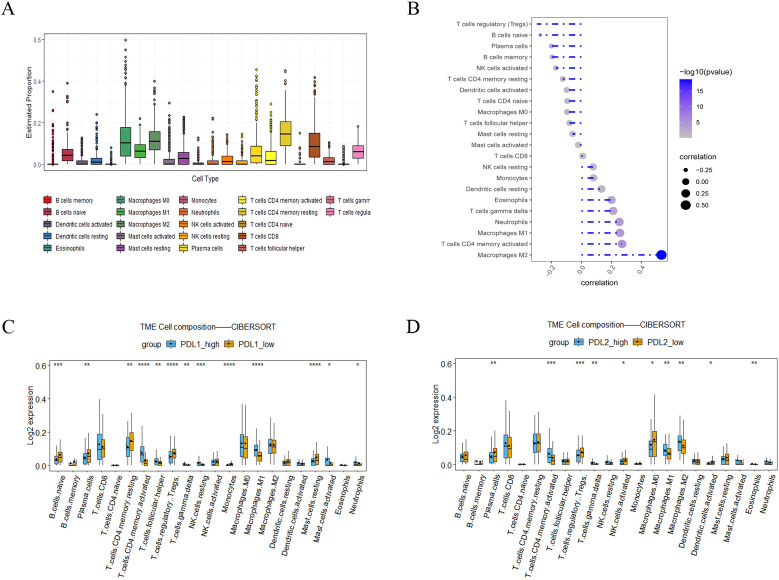
Correlations of LINC0104–PD-L1/PD-L2 with immune cell infiltration in GC. **(A)**, Immune cell abundance estimated by the CIBERSORT algorithm for 22 immune cell subsets. **(B)**, Relationship between LINC01094 expression and tumor immune cell infiltration proportions. **(C)**, Correlation matrix of immune cell content between the PD-L1 high- and low-expression group. **(D)**, Correlation matrix of immune cell content between the PD-L2 high- and low-expression group. *p < 0.05, **p < 0.01, ***p < 0.001 and ****p < 0.0001.

To understand the role of the LINC01094–PD-L1/PD-L2 axis in immune regulation in GC, we investigated the correlation of LINC01094 and PD-L1, as well as PD-L2, with immune cell infiltration. CIBERSORT showed the proportion of 22 immune cell subsets in the 375 TCGA-STAD samples, among which macrophages, CD4^+^ and CD8^+^ T cells are the main components of the immune microenvironment of gastric cancer tumors, (**Figure 7A**), indicating that these three types of cells play an important role in the occurrence and development of gastric tumors. Therefore, we further analyzed LINC01094, PD-L1 and PD-L2 with these cells. Spearman analysis indicated that LINC01094 expression level was positively correlated with M2 macrophages (**Figure 7B**, r = 0.47, *p* = 6.92E-22). Likewise, the high-PD-L2 group had a significantly higher proportion of M1 and M2 macrophages, as well as activated CD4^+^ memory T cells (**Figure 7C**). The activated CD4^+^ memory cells, helper T cells, M1 macrophages, resting NK cells and neutrophils were the most prevalent population in the high-PD-L1 expression group (**Figure 7D**). Of note, no significant associations were observed between the number of CD8^+^ T cells with PD-L1 (**Figure 7C**) and PD-L2 (**Figure 7D**), as well as LINC01094 (**Figure 7B**) expression (all *p* > 0.05).

### 3.8 LINC01094–PD-L2 is associated with CD8^+^ T cell dysfunction

**Figure 8:**
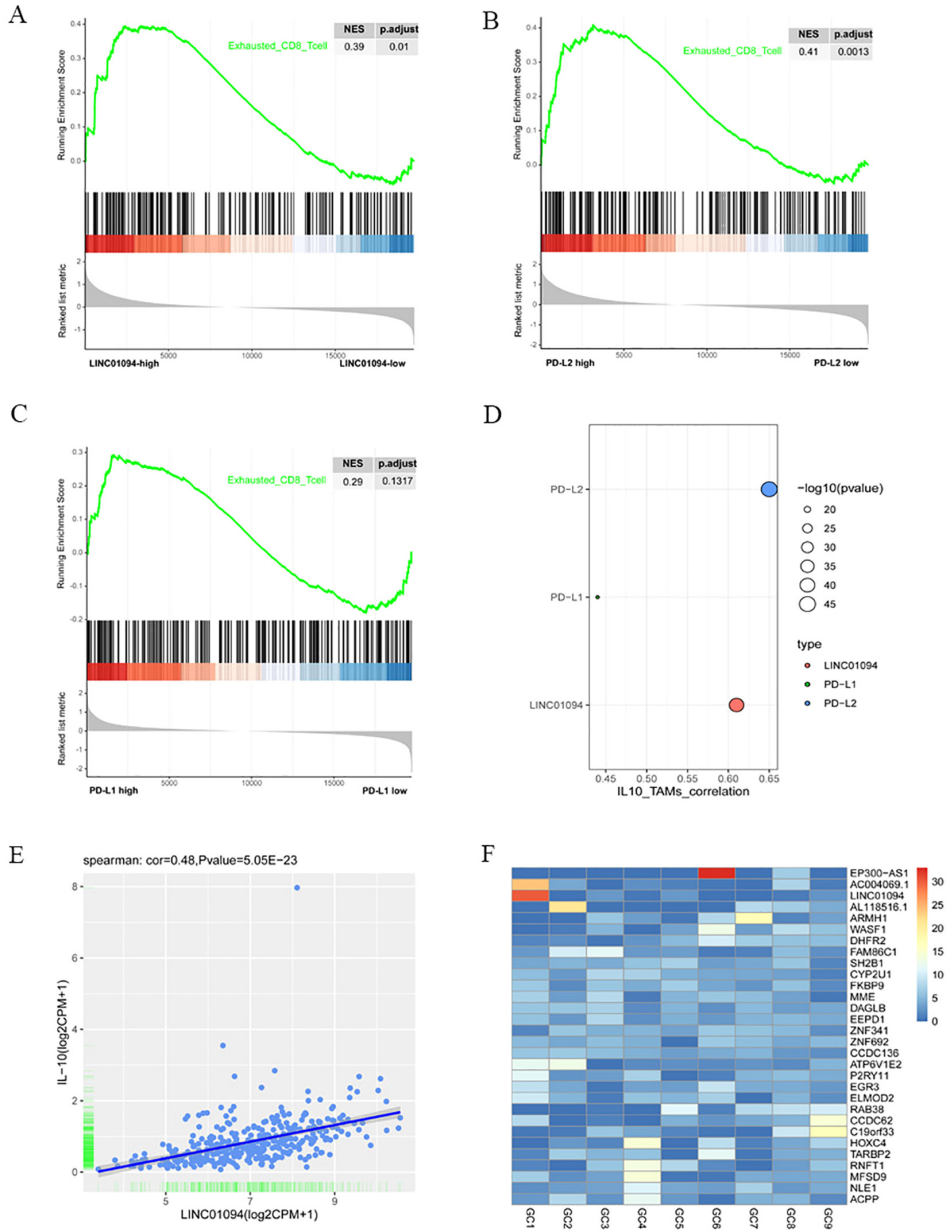
Associations of LINC01094–PD-L1/PD-L2 with CD8^+^ T cell dysfunction and IL-10^+^ TAMs. **(A)**, GSEA analysis of high-LINC01094 and low-LINC01094 group based on the genes related to CD8^+^ T cell dysfunction. **(B)**, GSEA analysis of high-PDL2 and low-PDL2 groups based on the genes related to CD8^+^ T cell dysfunction. **(C)**, GSEA analysis of high-PDL1 and low-PDL1 groups based on genes related to CD8^+^ T cell dysfunction. **(D)**, Correlations of IL-10^+^ TAMs with PDL1, PDL2 and LINC01094. **(E)**, Scatter plot of the correlation between the expression of LINC01094 and IL-10. **(F)**, Expression of lncRNAs in plasma exosomes of 9 individual GC patients.

It is well known that tumor-reactive CD8^+^ T cells are in a dysfunctional state during the course of tumorigenesis, with the term “exhaustion” being used to describe this phase (40). Therefore, we investigated the relationship between LINC01094, PD-L1, PD-L2 and functional status of CD8^+^ T cells by means of GSEA. The mRNA-seq data of 375 GC patients in TCGA were divided into high- and low-groups according to the median expression levels of LINC01094, PD-L1 and PD-L2. The results revealed that the CD8^+^ T cell dysfunction gene set was significantly up-regulated in the high-LINC01094 group (**Figure 8A**, NES = 0.39, *p.adjust* = 0.01), and high-PD-L2 group (**Figure 8B**, NES = 0.41, *p.adjust* = 0.0013). However, no significant association was found between the exhausted CD8^+^ T cell gene set and PD-L1 expression (**Figure 8C**, NES = 0.29, *p.adjust* = 0.13), suggesting that both LINC01094 and PD-L2, rather than PD-L1, contribute to orchestrate CD8^+^ T cell dysfunction in GC.

### 3.9 LINC01094–PD-L1/PD-L2 correlates with IL-10^+^ TAM infiltration

Since IL-10^+^ TAM infiltration has been revealed to yield regulatory T cell infiltration and CD8^+^ T cell dysfunction (37), we sought to find out the correlation between LINC01094–PD-L1/PD-L2 expression and the relative abundance of IL-10^+^ TAMs in the TCGA-STAD cohort. Using signature scores calculated by the geomean of expression counts of related genes, we found that PD-L1, PD-L2 and LINC01094 were positively correlated with IL-10^+^ TAM infiltration, the correlation coefficient was 0.44 (*p* = 3.83E-19), 0.65 (*p* = 4.28E-46) and 0.61 (*p* = 3.19E-40), respectively (**Figure 8D**).

Previous studies have documented that lncRNA can upregulate IL-10 expression and induce M2 macrophage polarization via competitively binding to miRNA (41–43). To explore a potential ceRNA mechanism of LINC01094 in regulating IL-10^+^ TAM infiltration, we analyzed the correlation between LINC01094 and IL-10 expression and discovered a positive correlation between LINC01094 and IL-10 expression in TCGA-STAD dataset (**Figure 8E**, r = 0.48, *p* = 5.05e-23). Then, using the Targrescan database, we predicted miR-17-5p also had potential binding sites for IL-10 (**Supplementary Figure 1**).

### 3.10 LINC01094 is present in exosomes of GC

Tumor-derived exosomal ncRNAs mediate the communication between tumor and immune cells, and contribute to M2 polarization (44). Assuming that LINC01094 is also transferred from exosomes from GC cells to macrophages, we investigated the expression level of LINC01094 using the exoRBase database. As **Supplementary Figure 4** showed that LINC01094 was exist in the exosomes of many cancers, and in some cancers with large sample sizes, such as breast cancer, pancreatic cancer and liver cancer. Although only 9 GC samples were included in the database, the data showed that LINC01094 was highly expressed in plasma exosomes of at least some GC patients (**Figure 8F**), suggesting that GC exosomal LINC01094 may be a potential mechanism underlying M2 macrophage polarization during tumor progression.

## 4 Discussion

Anti-PD-1 immunotherapies are believed to inhibit both PD-1/PD-L1 and PD-1/PD-L2 pathways simultaneously. However, immune-related adverse events associated with PD-1/PD-L1 blockade by ICIs, such as pneumonitis, are observed in clinical trials (45). Therefore, our finding of LINC01094 or miR-17-5p that could dual-targeting PD-L1 and PD-L2, rather than ICIs, might be a novel molecular target for immunotherapy with broad prospects. The lncRNA-associated ceRNA hypothesis is herein proposed to explain the regulatory mechanism for PD-L1 and PD-L2 in GC.

In the study, LINC01094 was identified as a hub lncRNA dual targeting both PD-L1 and PD-L2 in relation to poor prognosis and clinicopathological characteristics of GC. Based on the bioinformatics for miRNA binding sites and verified with dual-luciferase reporter assays, the LINC01094–miR-17-5p–PD-L1/PD-L2 interaction network was constructed. In view of the fact that both the LncATLAS data and *in vitro* cell experiments showed LINC01094 is mainly located in the cytoplasm (46, 47), we suggest that the dual targeting PD-L1 and PD-L2 of LINC01094 is due to LINC01094 acting as a ceRNA by sponging miR-17-5p. Furthermore, current data showed that LINC01094 expression was correlated with some clinicopathological features of GC, such as tumor stage, pathologic grade, and clinical stage, and EBV infection and MSI. It can thus be inferred that LINC01094 might act as a predictive and prognostic biomarker for GC patients, in particular for the specific molecular subtypes of EBV and MSI. This finding broadly supports the work of other studies linking LINC01094 with the progression of gliomas, glioblastoma, ovarian cancer, clear cell renal cell carcinoma, pancreatic cancer, colorectal cancer, and breast cancer (48–53). Additionally, A small molecule inhibitor targeting LINC01094 or miR-17-5p may be used as a potential immunotherapy strategy either alone or in combination with immunosuppressants.

The tumor immune microenvironment, comprising a vast variety of immune cells, has been shown to orchestrate tumor immunity and influence response to immune checkpoint blockade therapies (55). Generally, tumor-associated macrophages (TAMs) are the most abundant immune cells in solid tumors, consisting of antitumor M1-like and pro-tumor M2-like TAMs (56). The profiles of PD-L1 and PD-L2 in GC have been associated with immune cell infiltration (19). Consistent with the literature, we found that high expression of both PD-L1 and PD-L2 correlates with accumulation of activated memory CD4^+^ T cells and M1 macrophages. Interestingly, the present study only detected significant M2 macrophages accumulation in the high PD-L2 group, while no difference in enrichment for the M2 macrophage gene set was found between high- and low-PD-L1 groups, suggesting a difference in immunosuppression of the TME between PD-L1 and PD-L2. Similarly, we revealed that LINC0104 expression is strongly correlated with gene markers related to M2 macrophages, similar to that of Ye et al., who found that LINC01094 is correlated with macrophage infiltration in GC (54). It is worth noting that although no significant association was found between the number of CD8^+^ T cells and LINC01094, PD-L1 or PD-L2, the number of exhausted CD8^+^ T cells was high in the high LINC01094 and high PD-L2 groups. Together, these findings imply that PD-L2, rather than PD-L1, may play a primary role in LINC01094-induced M2 macrophage infiltration and CD8^+^ T cell dysfunction.

M2 polarization is a complex pathological process associated with immune evasion and poor prognosis in some malignancies (57). Various signaling pathways and multiple cytokines can drive M2 polarization, including IL-4, TGF-β, and IL-10 (58–61). Interestingly, when TAMs are polarized to be M2-like by IL-10, autocrine secretion of IL-10 increases, facilitating tumor growth, invasion and metastasis (62). In some cases, the secretion of IL-10 from TAMs is believed to induce PD-L1 upregulation in malignant cells (63). Consistently, a significant association between PD-L1 and IL-10^+^ TAM infiltration was confirmed in the present study, with associations between IL-10^+^ TAM and PD-L2 as well as LINC01094 being particularly strong. Moreover, the finding that LINC01094 has the highest degree of correlation with IL-10^+^ TAMs indicated that LINC01094 might be the most prominent regulator for the induction of IL-10^+^ TAM infiltration in GC. IL-10^+^ TAM infiltration in GC tissue has been proven to yield an immune escape tumor microenvironment featured by regulatory T cell infiltration and CD8^+^ T cell dysfunction. In particular, IL-10^+^ TAM infiltration is higher in EBV-positive tumors and PD-L1-positive cells (38). These existing findings provides rational explanation for the results of our work to link abnormal expression of LINC01094 with CD8^+^ T cell dysfunction. The field of CAR-T cell therapy is expanding swiftly and establishing a novel frontier in the treatment of hematological malignancies and other forms of cancer, however, hurdles persist due to immunosuppression and the poor ability of T cells to penetrate the tumor (64). Based on the findings of the current research, we hypothesized that CAR-T cells targeting LINC01094 might exhibit potentially enhanced toxicity against GC. Therefore, reduce the expression of LINC01094 may enhance the efficacy of CAR-T cells due to a more permissive microenvironment.

Exosome-encapsulated ncRNAs have been implicated in intercellular communication between tumor cells and TAMs, in which tumor-derived exosomal lncRNA-mediated M2 macrophage polarization is involved (44). For instance, colorectal cancer cell-derived exosomes transport lncRNA RPPH1 into macrophages to induce macrophage M2 polarization and promote colorectal cancer metastasis (65). Consistent with the literature, our data obtained from the exoRBase database suggested that LINC01094 really existed in plasma exosomes albeit differentially expressed among 9 GC patients. Interestingly, we found miR-17-5p have potential binding sites for IL-10. This result implied that LINC01094 might transported by exosomes from GC cells into macrophages, thereby sponging miR-17-5p to modulate IL-10 secretion. Therefore, plasma exosomal LINC01094 can be a novel biomarker for GC prognosis. However, the existence of a LINC01094/miR-17-5p/IL-10 axis in macrophages and its implication in macrophage polarization will be a focus of further investigation.

To the best of our knowledge, this is the first study to identify lncRNA dual-targeting of PD−L1 and PD−L2 and correlating with prognosis in GC. However, there are several limitations that should be noted. LINC01094-mediated crosstalk between IL-10^+^ TAMs CD8^+^ T cells and GC cells in tumor progression is not well understood, future validation experiments are then warranted to confirm the implication of LINC01094/miR-17-5p/IL-10 axis in shaping the immunosuppressive landscape, including macrophage polarization and CD8^+^ T cell dysfunction. Moreover, the available data regarding exosome-packaged LINC01094 in GC patients is limited. Further investigation on LINC01094 levels in GC cell-derived exosomes and exploration of its biological function are needed.

## 5 Conclusion

In summary, our findings suggest LINC01094 could dually targets PD-L1 and PD-L2 via sponging miR-17-5p, which might shape immunosuppressive tumor microenvironment in GC. Given the implications of this analysis, it is essential that future research focuses on examining the prognostic significance and exploring the therapeutic potential of LINC01094 in the context of GC immunotherapy.

## Data Availability

Publicly available datasets were analyzed in this study. This data can be found here: https://portal.gdc.cancer.gov/; https://www.ncbi.nlm.nih.gov/geo/query/acc.cgi?acc=GSE70880.

## Glossary

ceRNA: competing endogenous RNAs
circRNA: Circular RNA
CTLA-4: Cytotoxic T lymphocyte associated antigen 4
DElncRNAs: differentially expressed lncRNAs
EBV: Epstein-Barr virus
GC: Gastric cancer
GEO: Gene expression omnibus
GSEA: Gene set enrichment analysis
HER2: Human epidermal growth factor receptor. ICIs, Immune checkpoint inhibitors
IFN-γ: Interferon-γ
IL-10: Interleukin-10
IL-10^+^ TAM: IL-10 positive tumor-associated macrophages
LncRNA: Long Non-coding RNAs
mRNA: Messenger RNA
miRNA: MicroRNA
MSI: microsatellite instability
NK cell: Natural killer cell
OS: Overall survival
PD-1: Programmed cell death 1
PD-L1: Programmed cell death ligand 1
PD-L2: Programmed cell death ligand 2
STAD: Stomach adenocarcinoma
TAM: Tumor-associated macrophages
TCGA: The Cancer Genome Atlas
TGF-β: Transforming growth factor β
TME: Tumor microenvironment

## References

[1] Sung H Ferlay J Siegel RL Laversanne M Soerjomataram I Jemal A. Global cancer statistics 2020: GLOBOCAN estimates of incidence and mortality worldwide for 36 cancers in 185 countries. CA Cancer J Clin. (2021) 71:209–49. doi: 10.3322/caac.21660 33538338

[2] Kono K Nakajima S Mimura K. Current status of immune checkpoint inhibitors for gastric cancer. Gastric Cancer. (2020) 23:565–78. doi: 10.1007/s10120-020-01090-4 32468420

[3] Tang X Xu P Wang B Luo J Fu R Huang K. Peng H et al: Identification of a Specific Gene Module for Predicting Prognosis in Glioblastoma Patients. Front Oncol. (2019) 9:812. doi: 10.3389/fonc.2019.00812 31508371 PMC6718733

[4] Kundel Y Sternschuss M Moore A Perl G Brenner B Goldvaser H. Efficacy of immune-checkpoint inhibitors in metastatic gastric or gastroesophageal junction adenocarcinoma by patient subgroups: A systematic review and meta-analysis. Cancer Med. (2020) 9:7613–25. doi: 10.1002/cam4.3417 PMC7571828 32869544

[5] Zhou JT Liu JH Song TT Ma B Amidula N Bai C. EGLIF-CAR-T cells secreting PD-1 blocking antibodies significantly mediate the elimination of gastric cancer. Cancer Manag Res. (2020) 12:8893–902. doi: 10.2147/CMAR.S260915 PMC7520465 33061585

[6] Ding W LaPlant BR Call TG Parikh SA Leis JF He R. Feldman AL et al: Pembrolizumab in patients with CLL and Richter transformation or with relapsed CLL. Blood. (2017) 129:3419–27. doi: 10.1182/blood-2017-02-765685 PMC5492091 28424162

[7] Reck M Rodriguez-Abreu D Robinson AG Hui R Csoszi T Fulop A. Cuffe S et al: Pembrolizumab versus Chemotherapy for PD-L1-Positive Non-Small-Cell Lung Cancer. N Engl J Med. (2016) 375:1823–33. doi: 10.1056/NEJMoa1606774 27718847

[8] Fuchs CS Doi T Jang RW Muro K Satoh T MaChado M. Safety and Efficacy of Pembrolizumab Monotherapy in Patients With Previously Treated Advanced Gastric and Gastroesophageal Junction Cancer: Phase 2 Clinical KEYNOTE-059 Trial. JAMA Oncol. (2018) 4:e180013. doi: 10.1001/jamaoncol.2018.0013 29543932 PMC5885175

[9] Joshi SS Badgwell BD. Current treatment and recent progress in gastric cancer. CA Cancer J Clin. (2021) 71:264–79. doi: 10.3322/caac.21657 PMC9927927 33592120

[10] Dong Y Sun Q Zhang X. PD-1 and its ligands are important immune checkpoints in cancer. Oncotarget. (2017) 8:2171–86. doi: 10.18632/oncotarget.v8i2 PMC5356790 27974689

[11] Zak KM Grudnik P Magiera K Domling A Dubin G Holak TA. Structural biology of the immune checkpoint receptor PD-1 and its ligands PD-L1/PD-L2. Structure. (2017) 25:1163–74. doi: 10.1016/j.str.2017.06.011 28768162

[12] Qiao Y Liu C Zhang X Zhou Q Li Y Xu Y. Yang A et al: PD-L2 based immune signature confers poor prognosis in HNSCC. Oncoimmunology. (2021) 10:1947569. doi: 10.1080/2162402X.2021.1947569 34377590 PMC8344752

[13] Zhang Y Xu J Hua J Liu J Liang C Meng Q. A PD-L2-based immune marker signature helps to predict survival in resected pancreatic ductal adenocarcinoma. J immunotherapy Cancer. (2019) 7:233. doi: 10.1186/s40425-019-0703-0 PMC6716876 31464648

[14] Wang H Yao H Li C Liang L Zhang Y Shi H. PD-L2 expression in colorectal cancer: Independent prognostic effect and targetability by deglycosylation. Oncoimmunology. (2017) 6:e1327494. doi: 10.1080/2162402X.2017.1327494 28811964 PMC5543903

[15] Okadome K Baba Y Nomoto D Yagi T Kalikawe R Harada K. Iwatsuki M et al: Prognostic and clinical impact of PD-L2 and PD-L1 expression in a cohort of 437 oesophageal cancers. Br J Cancer. (2020) 122:1535–43. doi: 10.1038/s41416-020-0811-0 PMC7217865 32210369

[16] Shin SJ Jeon YK Kim PJ Cho YM Koh J Chung DH. Clinicopathologic analysis of PD-L1 and PD-L2 expression in renal cell carcinoma: association with oncogenic proteins status. Ann Surg Oncol. (2016) 23:694–702. doi: 10.1245/s10434-015-4903-7 26464193

[17] Nakayama Y Mimura K Kua LF Okayama H Min AKT Saito K. Momma T et al: Immune suppression caused by PD-L2 expression on tumor cells in gastric cancer. Gastric Cancer. (2020) 23:961–73. doi: 10.1007/s10120-020-01079-z 32367440

[18] Lingohr P Dohmen J Semaan A Branchi V Dietrich J Bootz F. Clinicopathological, immune and molecular correlates of PD-L2 methylation in gastric adenocarcinomas. Epigenomics. (2019) 11:639–53. doi: 10.2217/epi-2018-0149 30821175

[19] Liu J Li H Sun L Yuan Y Xing C. Profiles of PD-1, PD-L1, PD-L2 in gastric cancer and their relation with mutation, immune infiltration, and survival. BioMed Res Int. (2020) 2020:2496582. doi: 10.1155/2020/2496582 32596285 PMC7298268

[20] Liu X Choi MG Kim K Kim KM Kim ST Park SH. High PD-L1 expression in gastric cancer (GC) patients and correlation with molecular features. Pathol Res Pract. (2020) 216:152881. doi: 10.1016/j.prp.2020.152881 32089413

[21] Gu L Chen M Guo D Zhu H Zhang W Pan J. PD-L1 and gastric cancer prognosis: A systematic review and meta-analysis. PloS One. (2017) 12:e0182692. doi: 10.1371/journal.pone.0182692 28796808 PMC5552131

[22] Wu Y Cao D Qu L Cao X Jia Z Zhao T. PD-1 and PD-L1 co-expression predicts favorable prognosis in gastric cancer. Oncotarget. (2017) 8:64066–82. doi: 10.18632/oncotarget.v8i38 PMC5609984 28969052

[23] Fan F Chen K Lu X Li A Liu C Wu B. Dual targeting of PD-L1 and PD-L2 by PCED1B-AS1 via sponging hsa-miR-194-5p induces immunosuppression in hepatocellular carcinoma. Hepatol Int. (2020) 15:444–58. doi: 10.1007/s12072-020-10101-6 33219943

[24] Wang S Li XC Zhu JR Ma ZJ Ran JT Zhou YN. Construction of a novel ceRNA network and identification of lncRNA ADAMTS9-AS2 and PVT1 as hub regulators of miRNA and coding gene expression in gastric cancer. Trans Cancer Res. (2021) 10:938–52. doi: 10.21037/tcr PMC8799272 35116422

[25] Zhang G Li S Lu J Ge Y Wang Q Ma G. LncRNA MT1JP functions as a ceRNA in regulating FBXW7 through competitively binding to miR-92a-3p in gastric cancer. Mol Cancer. (2018) 17:87. doi: 10.1186/s12943-018-0829-6 29720189 PMC5930724

[26] Peng J Zhu Y Dong X Mao X Lou Y Mu Y. Construction and analysis of lncRNA-associated ceRNA network identified potential prognostic biomarker in gastric cancer. Trans Cancer Res. (2019) 8:1116–28. doi: 10.21037/tcr PMC8798625 35116854

[27] Zhang K Zhang L Mi Y Tang Y Ren F Liu B. A ceRNA network and a potential regulatory axis in gastric cancer with different degrees of immune cell infiltration. Cancer Sci. (2020) 111:4041–50. doi: 10.1111/cas.14634 PMC7648034 32860283

[28] Nie K Zheng Z Wen Y Pan J Liu Y Jiang X. Xu S et al: A novel ceRNA axis involves in regulating immune infiltrates and macrophage polarization in gastric cancer. Int Immunopharmacol. (2020) 87:106845. doi: 10.1016/j.intimp.2020.106845 32763781

[29] Wang L Cho KB Li Y Tao G Xie Z Guo B. (lncRNA)-mediated competing endogenous RNA networks provide novel potential biomarkers and therapeutic targets for colorectal cancer. Int J Mol Sci. (2019) 20:5758. doi: 10.3390/ijms20225758 PMC6888455 31744051

[30] Guo T Li J Zhang L Hou W Wang R Zhang J. Multidimensional communication of microRNAs and long non-coding RNAs in lung cancer. J Cancer Res Clin Oncol. (2019) 145:31–48. doi: 10.1007/s00432-018-2767-5 30417217 PMC11810324

[31] Nallasamy P Chava S Verma SS Mishra S Gorantla S Coulter DW. PD-L1, inflammation, non-coding RNAs, and neuroblastoma: Immuno-oncology perspective. Semin Cancer Biol. (2018) 52:53–65. doi: 10.1016/j.semcancer.2017.11.009 29196189 PMC5972043

[32] Li JH Liu S Zhou H Qu LH Yang JH. starBase v2.0: decoding miRNA-ceRNA, miRNA-ncRNA and protein-RNA interaction networks from large-scale CLIP-Seq data. Nucleic Acids Res. (2014) 42:D92–97. doi: 10.1093/nar/gkt1248 PMC3964941 24297251

[33] Shannon P Markiel A Ozier O Baliga NS Wang JT Ramage D. Cytoscape: a software environment for integrated models of biomolecular interaction networks. Genome Res. (2003) 13:2498–504. doi: 10.1101/gr.1239303 PMC403769 14597658

[34] Newman A Liu C Green M Gentles A Feng W Xu Y. Robust enumeration of cell subsets from tissue expression profiles. Nat. Methods. (2015) 12:453–7. doi: 10.1038/nmeth.3337 PMC4739640 25822800

[35] Hanzelmann S Castelo R Guinney J. GSVA: gene set variation analysis for microarray and RNA-seq data. BMC Bioinf. (2013) 14:7. doi: 10.1186/1471-2105-14-7 PMC3618321 23323831

[36] Subramanian A Tamayo P Mootha VK Mukherjee S Ebert BL Gillette MA. Lander ES et al: Gene set enrichment analysis: a knowledge-based approach for interpreting genome-wide expression profiles. Proc Natl Acad Sci United States America. (2005) 102:15545–50. doi: 10.1073/pnas.0506580102 PMC1239896 16199517

[37] Yu G Wang LG Han Y He QY. clusterProfiler: an R package for comparing biological themes among gene clusters. OMICS. (2012) 16:284–7. doi: 10.1089/omi.2011.0118 PMC3339379 22455463

[38] Zhang H Li R Cao Y Gu Y Lin C Liu X. Poor clinical outcomes and immunoevasive contexture in intratumoral IL-10-producing macrophages enriched gastric cancer patients. Ann Surg. (2020) 275:e626–35. doi: 10.1097/SLA.0000000000004037 32541216

[39] Cheng G Li Y Liu Z Song X. lncRNA PSMA3-AS1 promotes the progression of non-small cell lung cancer through targeting miR-17-5p/PD-L1. Adv Clin Exp Med. (2021) 30:1043–50. doi: 10.17219/acem/138624 34610219

[40] Philip M Schietinger A. CD8(+) T cell differentiation and dysfunction in cancer. Nat Rev Immunol. (2022) 22:209–23. doi: 10.1038/s41577-021-00574-3 PMC9792152 34253904

[41] Gao X Ge J Li W Zhou W Xu L. LncRNA KCNQ1OT1 ameliorates particle-induced osteolysis through inducing macrophage polarization by inhibiting miR-21a-5p. Biol Chem. (2018) 399:375–86. doi: 10.1515/hsz-2017-0215 29252185

[42] Ye M Xie M Zhu J Wang C Zhou R Li X. LPS-inducible lncRNA TMC3-AS1 negatively regulates the expression of IL-10. Front Immunol. (2020) 11:1418. doi: 10.3389/fimmu.2020.01418 32774335 PMC7387720

[43] Xu L Wang L Shi Y Deng Y Oates JC Kamen DL. Up-regulated interleukin-10 Induced by E2F transcription factor 2-MicroRNA-17-5p circuitry in extrafollicular effector b cells contributes to autoantibody production in systemic lupus erythematosus. Arthritis Rheumatol (Hoboken NJ). (2022) 74:496–507. doi: 10.1002/art.41987 PMC10403296 34569195

[44] Xu Z Chen Y Ma L Chen Y Liu J Guo Y. Role of exosomal non-coding RNAs from tumor cells and tumor-associated macrophages in the tumor microenvironment. Mol Ther. (2022) 30:3133–54. doi: 10.1016/j.ymthe.2022.01.046 PMC9552915 35405312

[45] Wang DY Johnson DB Davis EJ. Toxicities associated with PD-1/PD-L1 blockade. Cancer J (Sudbury Mass). (2018) 24:36–40. doi: 10.1097/PPO.0000000000000296 PMC5784852 29360726

[46] Dong X Fu X Yu M Li Z. Long intergenic non-protein coding RNA 1094 promotes initiation and progression of glioblastoma by promoting microRNA-577-regulated stabilization of brain-derived neurotrophic factor. Cancer Manag Res. (2020) 12:5619–31. doi: 10.2147/CMAR.S256147 PMC7359895 32765065

[47] Jiang Y Zhang H Li W Yan Y Yao X Gu W. FOXM1-activated LINC01094 promotes clear cell renal cell carcinoma development via microRNA 224-5p/CHSY1. Mol Cell Biol. (2020) 40:e00357–19. doi: 10.1128/MCB.00357-19 PMC6965037 31767633

[48] Li XX Yu Q. Linc01094 accelerates the growth and metastatic-related traits of glioblastoma by sponging miR-126-5p. Onco Targets Ther. (2020) 13:9917–28. doi: 10.2147/OTT.S263091 PMC7547807 33116576

[49] Xu J Zhang P Sun H Liu Y. LINC01094/miR-577 axis regulates the progression of ovarian cancer. J Ovarian Res. (2020) 13:122. doi: 10.1186/s13048-020-00721-9 33069244 PMC7568364

[50] Jiang Y Li W Yan Y Yao X Gu W Zhang H. LINC01094 triggers radio-resistance in clear cell renal cell carcinoma via miR-577/CHEK2/FOXM1 axis. Cancer Cell Int. (2020) 20:274. doi: 10.1186/s12935-020-01306-8 32595418 PMC7315499

[51] Luo C Lin K Hu C Zhu X Zhu J Zhu Z. LINC01094 promotes pancreatic cancer progression by sponging miR-577 to regulate LIN28B expression and the PI3K/AKT pathway. Mol Ther Nucleic Acids. (2021) 26:523–35. doi: 10.1016/j.omtn.2021.08.024 PMC8479296 34631282

[52] Zhang G Gao Y Yu Z Su H. Upregulated long intergenic non-protein coding RNA 1094 (LINC01094) is linked to poor prognosis and alteration of cell function in colorectal cancer. Bioengineered. (2022) 13:8526–37. doi: 10.1080/21655979.2022.2051839 PMC9161846 35287563

[53] Wu X Kong C Wu Y. Long intergenic non-protein coding RNA 1094 (LINC01094) promotes the progression of breast cancer (BC) by regulating the microRNA-340-5p (miR-340-5p)/E2F transcription factor 3 (E2F3) axis. Bioengineered. (2021) 12:9046–57. doi: 10.1080/21655979.2021.1993715 PMC8806954 34657558

[54] Ye Y Ge O Zang C Yu L Eucker J Chen Y. LINC01094 predicts poor prognosis in patients with gastric cancer and is correlated with EMT and macrophage infiltration. Technol Cancer Res Treat. (2022) 21:15330338221080977. doi: 10.1177/15330338221080977 35254147 PMC8905065

[55] Fu T Dai LJ Wu SY Xiao Y Ma D Jiang YZ. Spatial architecture of the immune microenvironment orchestrates tumor immunity and therapeutic response. J Hematol Oncol. (2021) 14:98. doi: 10.1186/s13045-021-01103-4 34172088 PMC8234625

[56] Pan Y Yu Y Wang X Zhang T. Tumor-associated macrophages in tumor immunity. Front Immunol. (2020) 11:583084. doi: 10.3389/fimmu.2020.583084 33365025 PMC7751482

[57] Najafi M Hashemi Goradel N Farhood B Salehi E Nashtaei MS Khanlarkhani N. Macrophage polarity in cancer: A review. J Cell Biochem. (2019) 120:2756–65. doi: 10.1002/jcb.27646 30270458

[58] Liu Q Yang C Wang S Shi D Wei C Song J. Wnt5a-induced M2 polarization of tumor-associated macrophages via IL-10 promotes colorectal cancer progression. Cell Commun Signal. (2020) 18:51. doi: 10.1186/s12964-020-00557-2 32228612 PMC7106599

[59] Batlle E Massague J. Transforming growth factor-beta signaling in immunity and cancer. Immunity. (2019) 50:924–40. doi: 10.1016/j.immuni.2019.03.024 PMC7507121 30995507

[60] Ruffell B Chang-Strachan D Chan V Rosenbusch A Ho CM Pryer N. Macrophage IL-10 blocks CD8+ T cell-dependent responses to chemotherapy by suppressing IL-12 expression in intratumoral dendritic cells. Cancer Cell. (2014) 26:623–37. doi: 10.1016/j.ccell.2014.09.006 PMC4254570 25446896

[61] Huang X Li Y Fu M Xin HB. Polarizing macrophages. In Vitro. Methods Mol Biol. (2018) 1784:119–26. doi: 10.1007/978-1-4939-7837-3_12 PMC8875934 29761394

[62] Boutilier AJ Elsawa SF. Macrophage polarization states in the tumor microenvironment. Int J Mol Sci. (2021) 22:6995. doi: 10.3390/ijms22136995 PMC8268869 34209703

[63] Kuang DM Zhao Q Peng C Xu J Zhang JP Wu C. Activated monocytes in peritumoral stroma of hepatocellular carcinoma foster immune privilege and disease progression through PD-L1. J Exp Med. (2009) 206:1327–37. doi: 10.1007/978-1-4939-7837-3_12 PMC2715058 19451266

[64] Khan TH Muhammad N Tarique M Usmani D Naz H Sarode A. The role of cancer-specific target antigens in CAR T cell therapy in hematological Malignancies. Curr Tissue Microenviron Rep. (2024) 5:61–7. doi: 10.1007/s43152-024-00055-4

[65] Liang ZX Liu HS Wang FW Xiong L Zhou C Hu T. LncRNA RPPH1 promotes colorectal cancer metastasis by interacting with TUBB3 and by promoting exosomes-mediated macrophage M2 polarization. Cell Death Dis. (2019) 10:829. doi: 10.1038/s41419-019-2077-0 31685807 PMC6828701

