# Supplementary material for: Identification of lncRNA dual targeting PD-L1 and PD-L2 as a novel prognostic predictor for gastric cancer
Source: Front Oncol. 2024 Oct 25;14:1341056. doi: 10.3389/fonc.2024.1341056 (PMC11544118; doi:10.3389/fonc.2024.1341056)
Supplement: Supplementary file 1 [file DataSheet1.docx]

**Supplementary Table 1.** Primers of LINC01094, miR-17-5p, PD-L1, PD-L2 and β-actin.

| Primer | Sense (5' to 3') | Anti-sense (5' to 3') |
| --- | --- | --- |
| LINC01094 | GAAGTCAGAGTGTTGCTTGCC | TAGCGTGCCTAAATCGAATCCG |
| PD-L1 | GCTATGGTGGTGCCGACTAC | TGGCTCCCAGAATTACCAAGT |
| PD-L2 | ACCGTGAAAGAGCCACTTTG | GCGACCCCATAGATGATTATGC |
| β-actin | AGCGAGCATCCCCCAAAGTT | GGGCACGAAGGCTCATCATT |

**Supplementary Table 2.** The sequences of LINC01094 siRNA, miR-17-5p mimics and inhibitors.

|  | Sense (5' to 3') | Anti-sense (5' to 3') |
| --- | --- | --- |
| LINC01094 siRNA#1 | GGUGGUUUCCCAAUCUUAATT | UUAAGAUUGGGAAACCACCTT |
| LINC01094 siRNA#2 | GGAUAUGGACUCCUUCCAATT | UUGGAAGGAGUCCAUAUCCTT |

| **Supplementary Table 3.** Gene list of IL-10^+^ TAM signature and the exhausted CD8^+^ T cell gene set | |
| --- | --- |
| **Gene sets** | **Genes** |
| **IL10^+^ TAM signature** | IL10, STAT3, MAF, MAPK3, TLR9, STAT1, CD68, CD163, VCAM1, IRF3, CD81 |
| **Exhausted CD8^+^ T cell** | ABCG1, ACADVL, ACSL1, ADAM7, AFP, AGAP1, AHR, ANXA3, APP, ATP2A2, ATP5J2, AUH, BET1, C10orf58, C12orf41, C16orf72, C17orf79, C3orf78, C8orf85, C9orf114, C9orf3, CADM1, CANX, CARM1, CCRL2, CD22, CD244, CELA1, CELF4, CFH, CFHR2, CHL1, CKMT2, CLCA1, CLDN11, COCH, COL19A1, CPA3, CPSF2, CRISP2, CSF1, CXCL13, CXCL14, CXorf26, CYP2A6, CYP4V2, DDIT4, DFFA, DOCK7, DPP7, DUSP6, EFNB3, EFS, EGR2, EIF2AK2, ENPP2, EOMES, EPCAM, ERCC5, EVI5, EXOSC8, F2RL1, FAM101B, FAM207A, FGF6, FHL1, FRK, GABRR2, GATA2, GCET2, GCM2, GDAP1, GDNF, GMCL1, GNAO1, GPLD1, GPM6B, GPR56, GSTM3, GSTO1, GTF3C4, H19, HAO2, HINFP, HIST1H1E, HIST1H3F, HLA-DMA, HMGA2, HOXC6, HTRA2, IFIH1, IGF1R, IL1A, IMMT, INCA1, IRF6, IRS1, KCNAB1, KCTD12, KIAA1217, LCLAT1, LHCGR, LIN9, LOC81691, MAGEL2, MAP1S, MAP2, MCAM, MDN1, METAP2, MITF, MRPL48, MRPS2, MSX1, MTRF1L, MYO6, NAP1L2, NCAN, NDUFA13, NEFH, NEUROD1, NFIB, NKIRAS1, NKX2-2, NOTCH4, NQO1, NR4A2, NRK, NSDHL, OVGP1, P2RX4, PAWR, PAX1, PCLO, PER2, PHLDA3, PHLDB2, PIK3C2G, PLA2G10, PLEKHA1, PNRC1, POLR1B, POLR2C, POU2F1, PTGER2, PTK6, PTPN12, PTPRJ, RBM15, RDBP, RGS10, RGS16, RHAG, RIN2, RPRD1B, SCAMP1, SCAND1, SCN1A, SCN7A, SCRG1, SIX1, SLC12A2, SLC30A1, SLC6A4, SLC7A11, SMAD1, SPOCK2, SPP1, SPRED2, SSBP2, STRA6, SUB1, SYT1, TAPBP, TBX15, TCF4, TERF1, TGM2, TLR7, TM2D1, TM2D3, TM4SF1, TMEM150A, TMEM5, TNFRSF4, TNFRSF9, TRPC1, TUG1, TWSG1, VAMP7, VCAM1, WFS1, WLS, YAP1, ZFP28, ZNF239, ZNF35, ZNF821, ZNRF1, ZRANB1 |

**Supplementary Table 4.** Basic clinical features of the TCGA-STAD cohort

|  | Alive  (N=228) | Dead  (N=147) | Overall  (N=375) |
| --- | --- | --- | --- |
| Age (years) |  |  |  |
| Mean (SD) | 65.1 (11.1) | 66.9 (9.85) | 65.8 (10.7) |
| Median [Min, Max] | 66.0 [35.0, 90.0] | 68.0 [41.0, 90.0] | 67.0 [35.0, 90.0] |
| Missing | 4 (1.8%) | 0 (0%) | 4 (1.1%) |
| Sex |  |  |  |
| Male | 139 (61.0%) | 102 (69.4%) | 241 (64.3%) |
| Female | 89 (39.0%) | 45 (30.6%) | 134 (35.7%) |
| Pathologic_Stage |  |  |  |
| Stage I | 41 (18.0%) | 12 (8.2%) | 53 (14.1%) |
| Stage II | 77 (33.8%) | 34 (23.1%) | 111 (29.6%) |
| Stage III | 82 (36.0%) | 68 (46.3%) | 150 (40.0%) |
| Stage IV | 15 (6.6%) | 23 (15.6%) | 38 (10.1%) |
| Missing | 13 (5.7%) | 10 (6.8%) | 23 (6.1%) |
| Pathologic_T |  |  |  |
| T1 | 17 (7.5%) | 2 (1.4%) | 19 (5.1%) |
| T2 | 53 (23.2%) | 27 (18.4%) | 80 (21.3%) |
| T3 | 95 (41.7%) | 73 (49.7%) | 168 (44.8%) |
| T4 | 59 (25.9%) | 41 (27.9%) | 100 (26.7%) |
| TX | 4 (1.8%) | 4 (2.7%) | 8 (2.1%) |
| Pathologic_M |  |  |  |
| M0 | 204 (89.5%) | 126 (85.7%) | 330 (88.0%) |
| M1 | 11 (4.8%) | 14 (9.5%) | 25 (6.7%) |
| MX | 13 (5.7%) | 7 (4.8%) | 20 (5.3%) |
| Pathologic_N |  |  |  |
| N0 | 82 (36.0%) | 29 (19.7%) | 111 (29.6%) |
| N1 | 56 (24.6%) | 41 (27.9%) | 97 (25.9%) |
| N2 | 46 (20.2%) | 30 (20.4%) | 76 (20.3%) |
| N3 | 32 (14.0%) | 42 (28.6%) | 74 (19.7%) |
| NX | 11 (4.8%) | 5 (3.4%) | 16 (4.3%) |
| Missing | 1 (0.4%) | 0 (0%) | 1 (0.3%) |
| Histological_Grade |  |  |  |
| G1 | 8 (3.5%) | 2 (1.4%) | 10 (2.7%) |
| G2 | 88 (38.6%) | 49 (33.3%) | 137 (36.5%) |
| G3 | 127 (55.7%) | 92 (62.6%) | 219 (58.4%) |
| GX | 5 (2.2%) | 4 (2.7%) | 9 (2.4%) |
| MSI |  |  |  |
| No | 181 (79.4%) | 105 (71.4%) | 286 (76.3%) |
| Yes | 36 (15.8%) | 25 (17.0%) | 61 (16.3%) |
| Missing | 11 (4.8%) | 17 (11.6%) | 28 (7.5%) |
| EBV |  |  |  |
| No | 203 (89.0%) | 117 (79.6%) | 320 (85.3%) |
| Yes | 14 (6.1%) | 13 (8.8%) | 27 (7.2%) |
| Missing | 11 (4.8%) | 17 (11.6%) | 28 (7.5%) |

**Supplementary Table 5.** Identification of lncRNAs correlated with PD-L1 and PD-L2 by spearman analysis

| **mRNA** | **lncRNAs** |
| --- | --- |
| **PD-L1** | RP11-44K6.2, RP11-44K6.4, AC002331.1, CTD-2288O8.1, AC133644.2,  LINC01094, AC112721.2, CTB-114C7.4, AC145110.1, RP5-1028K7.2,  RP11-322D14.2, AC004988.1, SIRPG-AS1, AC017002.1, RP11-79H23.3  RP11-1008C21.1, LINC00877, RP11-212I21.2, RP11-1149M10.2, LINC01272,  CTA-384D8.31, CTD-3195I5.4, CTD-2023M8.1, RP11-356I2.1, CTA-384D8.35  RP11-1018N14.5, PIK3CD-AS1, RP11-556E13.1, HCP5 |
| **PD-L2** | LINC01094, AC133644.2, LINC00877, AC079767.4, PIK3CD-AS1, SENCR,  CTB-114C7.4, AC004988.1, AC002331.1, RP11-212I21.2, SIRPG-AS1,  LINC01229, AC112721.2, CTD-3128G10.7, AC017002.1, CTD-2288O8.1,  MAGI2-AS3, RP11-452C13.1, AP001046.5, RP11-733O18.1, AC145110.1,  RP11-1018N14.5, AF131217.1, RP11-875O11.1, RP11-44K6.2, RP5-1028K7.2,  LINC01550, RP11-44K6.4, AC079630.4, RP4-575N6.4, RP11-1070N10.3,  RP11-1149M10.2, AC007750.5, RP11-344B5.2, LINC00519, AL122127.25,  RP11-536K7.5, RP11-426C22.4, RP11-556E13.1, LINC01059, AC104699.1,  RP11-356N1.2, AC006272.2, RP11-532F6.3, RP4-598P13.1, BFSP2-AS1,  AC093850.2, RP11-710C12.1, CTD-3035K23.7, RP11-166D19.1, RP11-400K9.4,  LINC01272, RP11-212I21.5, RP11-276H19.1, AC010226.4, LINC00582,  ST3GAL6-AS1, RP11-1008C21.1, RP11-89K11.1, AC104654.2, VCAN-AS1,  CTD-2089N3.1, MEF2C-AS1, RP11-400K9.3, RP11-586D19.1, NR2F1-AS1,  RP5-965F6.2, CTD-2616J11.3, RP11-322D14.2, LINC01140, RP11-145M4.3,  AC108463.1, PGM5P3-AS1, INHBA-AS1, AC096579.13, RP11-13P5.2,  RP11-576I22.2, FAM225A, RP11-109E24.1, MIR100HG, RP11-736K20.5,  RP11-444D3.1, RP11-443C10.1, LINC00152, AC010095.5 |

**
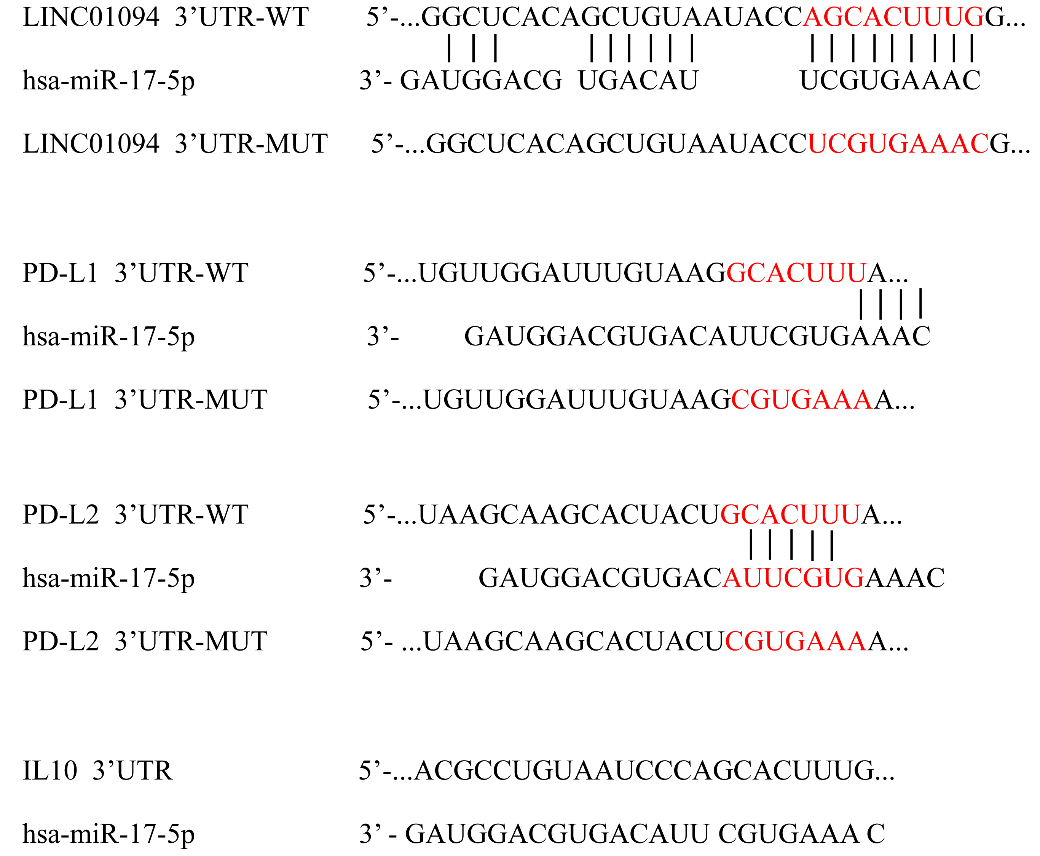
**

**Supplementary Figure 1.** The schematics of the WT binding site and MUT sequence of **(A)** LINC01094 and miR-17-5p, **(B)** PD-L1 and miR-17-5p, **(C)** PD-L2 and miR-17-5p. **D,** Binding sites between IL-10 and miR-17-5p was predicted by TargetScan.


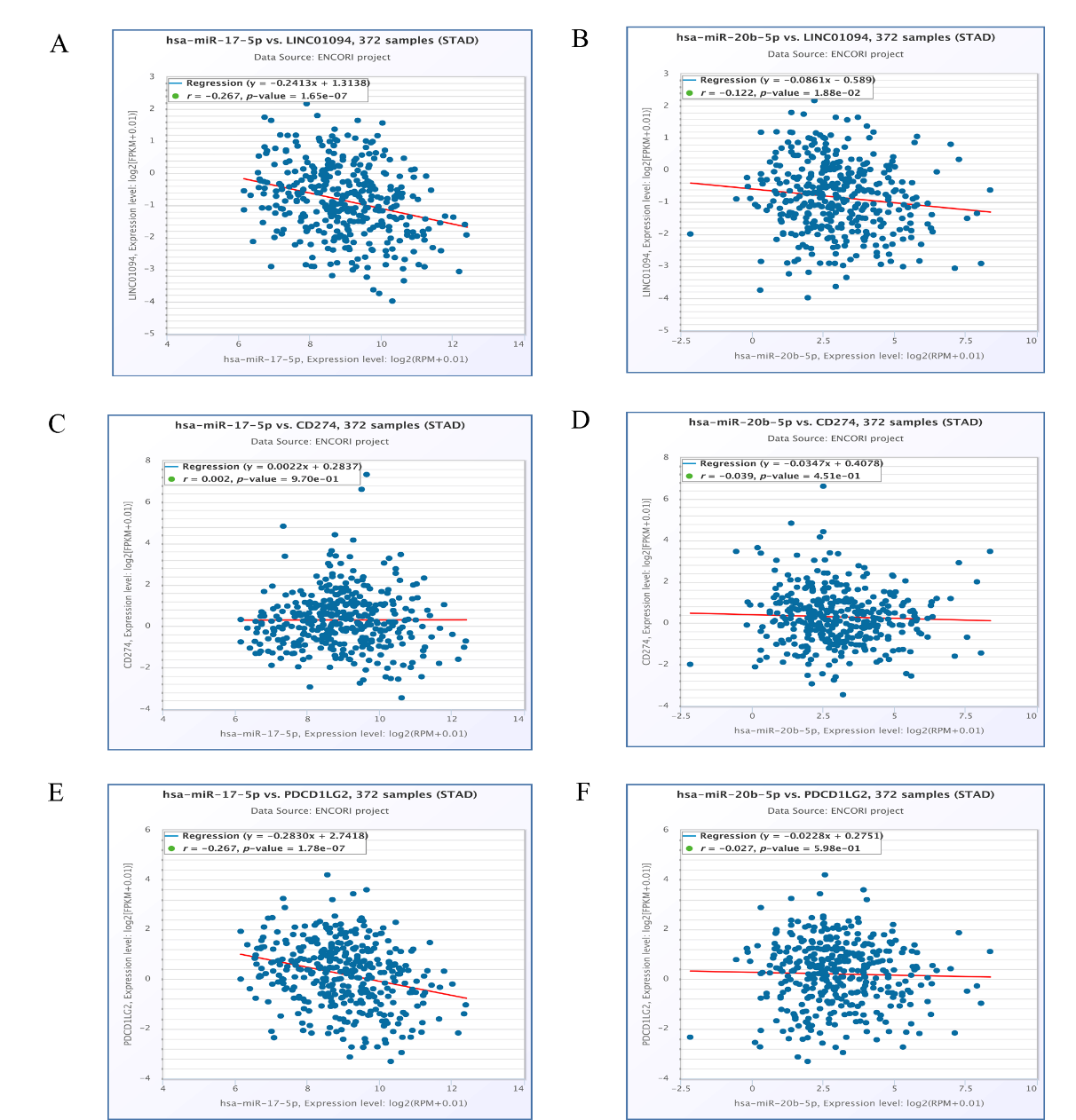


**Supplementary Figure 2.** Pearson correlation analysis between LINC01094, mRNA levels of PD-L1 and PD-L2 and candidate miRNAs in GC (data came from ENCORI). Correlations of **(A)** LINC01094 with miR-17-5p and **(B)** miR-20b-5p**.** Correlations of PD-L1 with **(C)** miR-17-5p and **(D)** miR-20b-5p**.** Correlations of PD-L2 with **(E)** miR-17-5p and **(F)** miR-20b-5p**.**


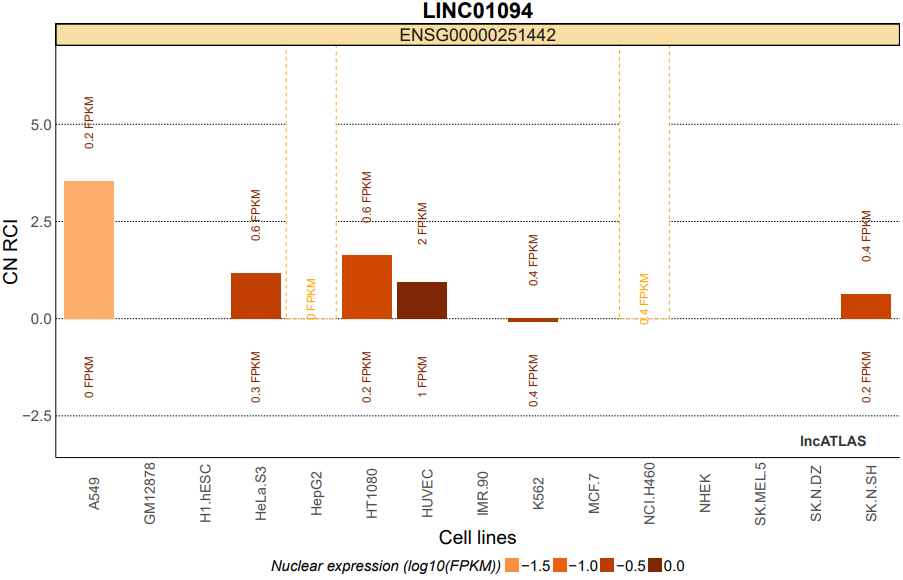


**Supplementary Figure 3.** Subcellular localization plots of LINC01094 displayed by LncATLAS.


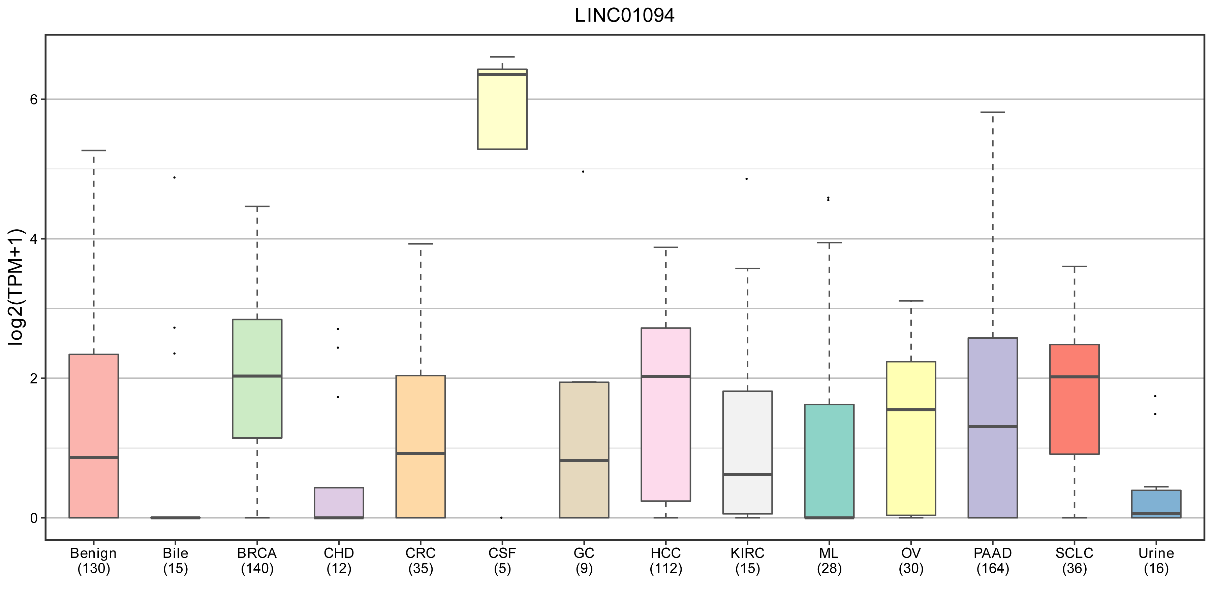


**Supplementary Figure 4.** LINC01094 expression in different tumors in the exoRBase database.
